# Carbonate-promoted C–H carboxylation of electron-rich heteroarenes†

**DOI:** 10.1039/d0sc04548a

**Published:** 2020-10-05

**Authors:** Tyler M. Porter, Matthew W. Kanan

**Affiliations:** Department of Chemistry, Stanford University Stanford California 94305 USA mkanan@stanford.edu

## Abstract

C–H carboxylation is an attractive transformation for both streamlining synthesis and valorizing CO_2_. The high bond strength and very low acidity of most C–H bonds, as well as the low reactivity of CO_2_, present fundamental challenges for this chemistry. Conventional methods for carboxylation of electron-rich heteroarenes require very strong organic bases to effect C–H deprotonation. Here we show that alkali carbonates (M_2_CO_3_) dispersed in mesoporous TiO_2_ supports (M_2_CO_3_/TiO_2_) effect CO_3_^2−^-promoted C–H carboxylation of thiophene- and indole-based heteroarenes in gas–solid reactions at 200–320 °C. M_2_CO_3_/TiO_2_ materials are strong bases in this temperature regime, which enables deprotonation of very weakly acidic bonds in these substrates to generate reactive carbanions. In addition, we show that M_2_CO_3_/TiO_2_ enables C3 carboxylation of indole substrates *via* an apparent electrophilic aromatic substitution mechanism. No carboxylations take place when M_2_CO_3_/TiO_2_ is replaced with un-supported M_2_CO_3_, demonstrating the critical role of carbonate dispersion and disruption of the M_2_CO_3_ lattice. After carboxylation, treatment of the support-bound carboxylate products with dimethyl carbonate affords isolable esters and the M_2_CO_3_/TiO_2_ material can be regenerated upon heating under vacuum. Our results provide the basis for a closed cycle for the esterification of heteroarenes with CO_2_ and dimethyl carbonate.

## Abstract 2

Carboxylation of heteroarenes with CO_2_ is achieved using alkali carbonate dispersed in mesoporous titania as a regenerable reagent.

## Graphical abstract

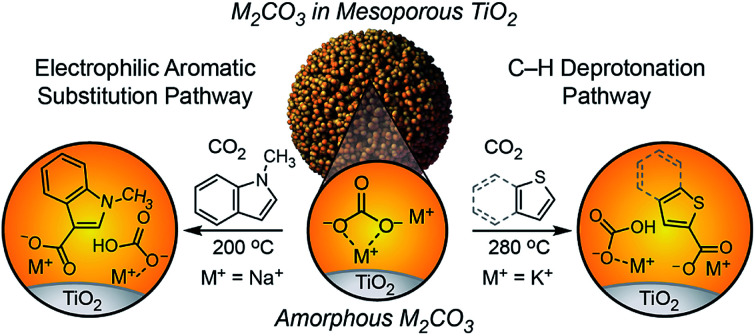

## Introduction

**Scheme 1:**
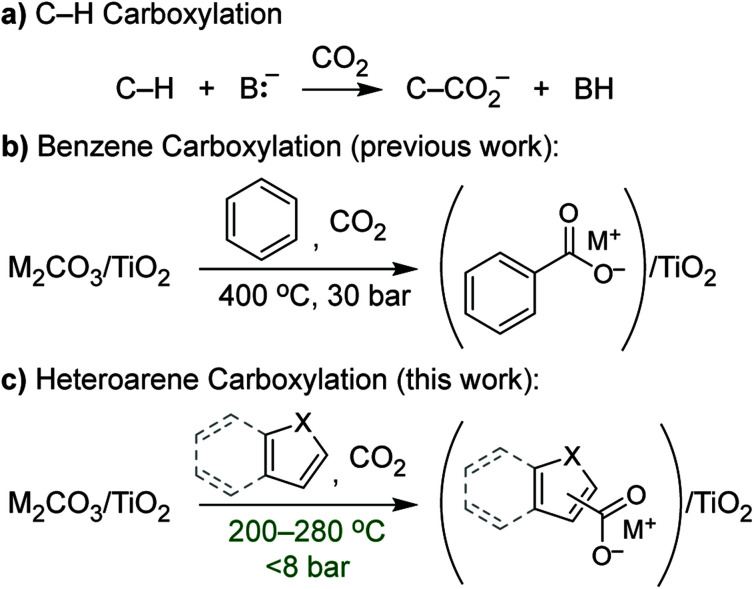
(a) General scheme for base-promoted C–H carboxylation; (b) C–H carboxylation of benzene using dispersed alkali carbonates (M_2_CO_3_/TiO_2_); (c) C–H carboxylation of heteroarenes using M_2_CO_3_/TiO_2_.

C–H carboxylation (Scheme 1) is a compelling alternative to conventional syntheses of carboxylic acids that utilize oxidative transformations or more functionalized substrates and has attracted attention as a way to expand the use of CO_2_ in chemical production.^1–3^ However, carboxylation faces the challenge of overcoming the low reactivity of C–H bonds and CO_2_, and it lacks the large intrinsic driving force of other C–H functionalizations such as oxidation or amination. The insertion of CO_2_ into C–H bonds to form a carboxylic acid is actually endergonic on account of the small Δ*H* and negative Δ*S*, while C–H carboxylation is exergonic (depending on base strength) because of the driving force from deprotonation. Increased interest in this transformation over the last several years has led to a number of methods that encompass both acid–base^2,4–9^ (ionic) and radical mechanisms^2,10–14^ for C–H activation. Despite these recent advances, most methods for C–H carboxylation under conventional, solution-phase conditions require highly reactive, resource-intensive reagents to activate C–H bonds. As such, the development of alternatives that use benign, regenerable reagents is critical to create opportunities for scalable CO_2_ utilization.

The carboxylation of aromatic substrates is of particular interest for the synthesis of a wide variety of both fine and commodity chemicals.^2,15–19^ Because of the high bond dissociation enthalpy (BDE) of aromatic C–H bonds, acid–base (ionic) activation of the substrate has been the most commonly employed strategy. For some substrates, deprotonation of an X–H bond (X = heteroatom) generates a nucleophilic intermediate that undergoes C–H carboxylation *via* an electrophilic aromatic substitution (EAS) mechanism. The classic example is the Kolbe–Schmidt reaction used for aspirin synthesis, in which phenol is transformed into salicylate by reaction with hydroxide and CO_2_.^20^ While the carboxylation of indoles and pyrroles has been achieved similarly,^6,8,21^ these reactions have required the use of superstoichiometric LiO^*t*^Bu to deprotonate the N–H bonds.

Apart from these special cases, carboxylation of (hetero)arene substrates *via* acid–base chemistry requires direct activation of the C–H bond to generate a reactive carbon-centered nucleophile. Within the last decade, several groups have demonstrated Brønsted-base-promoted carboxylation of (hetero)arenes in organic solvents at near ambient CO_2_ pressure.^4,6–9,21^ Hu *et al.* have shown that relatively acidic heteroarenes (p*K*_a_ up to 28 in organic solvent) can be carboxylated using Cs_2_CO_3_ as the base in refluxing DMF.^4,7^ The carboxylation of electron-rich heteroarenes beyond this p*K*_a_ threshold, however, has required much stronger bases. For example, the carboxylation of benzothiophene (p*K*_a_ of 33 in THF) was not possible under these same conditions.^4^ Recently, Kondo *et al.* have demonstrated the carboxylation of a diverse set of (benzo)thiophenes and (benzo)furans by reaction with excess LiO^*t*^Bu, CsF, and crown ether at 160 °C under a CO_2_ atmosphere.^9^ However, these conditions were not able to carboxylate protected indoles, such as 1-methylindole, whose C2 carbon has a p*K*_a_ near 38. Carboxylation of electron rich heteroarenes functionalized with an amide directing group has been achieved using Ni catalysis with stoichiometric KO^*t*^Bu and Mn^0^.^22^

Arenes have p*K*_a_s that generally lie beyond what can be measured (p*K*_a_ > 40). Researchers have developed methods to carboxylate arenes that are functionalized with a directing group by using a Rh or Pd species to catalyze C–H activation.^23–25^ In addition to the directing group and catalyst, these methods also require a strong base (KO^*t*^Bu) or Lewis acid activator (AlMe(OMe)_2_) to engender reactivity. In the absence of a directing group, solution-phase arene C–H carboxylation requires an extremely strong base such as Schlosser's base,^26^ or stoichiometric aluminum reagents.^25,27^

Apart from acid–base strategies, a very recent report by König *et al.* has described a photoredox method to carboxylate of (hetero)arenes under mild conditions in which the substrate is activated by one-electron photoreduction and Cs_2_CO_3_ serves as the stoichiometric base.^28^ This method affords moderate to high yields across a variety of substrates, although it is presently incompatible with some classes of (hetero)arene substrates and uses relatively high loadings of a photocatalyst requiring multi-step synthesis.

**Fig. 1:**
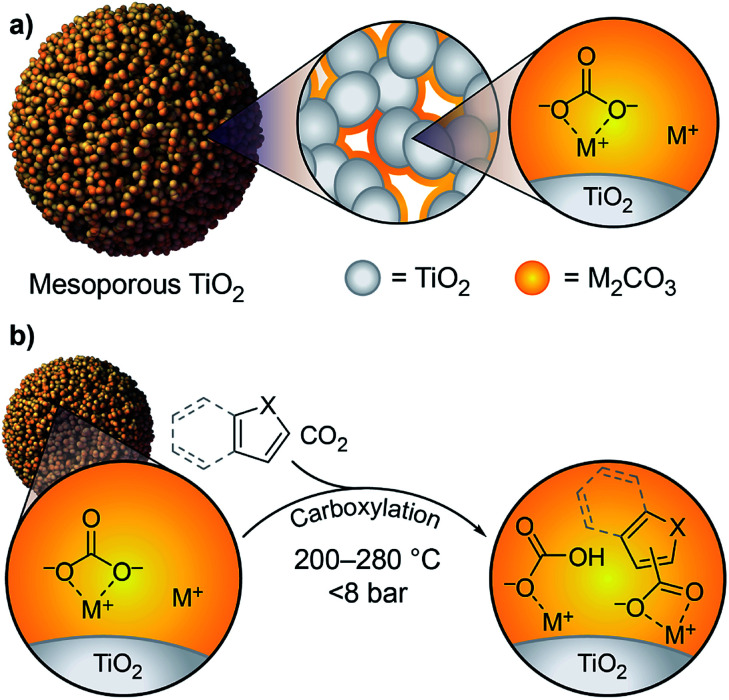
(a) Schematic view of M_2_CO_3_/TiO_2_ showing the M_2_CO_3_ dispersed over the mesoporous TiO_2_ surface; (b) heteroarene C–H carboxylation promoted by M_2_CO_3_/TiO_2_. Note that MHCO_3_ is shown for simplicity, but under the reaction conditions it is thermally unstable and will convert to 0.5 equiv. M_2_CO_3_, H_2_O, and CO_2_.

We previously showed that simple alkali carbonates (M_2_CO_3_) can promote C–H carboxylation of very weakly acidic substrates in solvent-free, alkali salt media at elevated temperature.^29–32^ This transformation is particularly useful for converting a monocarboxylate substrate into a dicarboxylate product, where the substrate enables the formation of a molten reaction medium.^33^ More recently, we demonstrated that M_2_CO_3_ dispersed into mesoporous TiO_2_ (M_2_CO_3_/TiO_2_, Fig. 1a) promotes the carboxylation of benzene and other aromatic hydrocarbon C–H bonds in gas–solid reactions (Scheme 1b).^34^ Dispersion in mesopores disrupts the bulk M_2_CO_3_ crystal structure, creating an amorphous material that can attain superbase reactivity, even in the presence of CO_2_. This carbonate-promoted C–H carboxylation of aromatic hydrocarbons takes place at moderate pressures and temperatures of ∼400 °C. In this study, we begin to assess the generality and selectivity of this strategy using electron-rich heteroarenes, which have somewhat more acidic C–H bonds. We show that gas–solid carbonate-promoted C–H carboxylation occurs at substantially lower temperatures for these substrates and that selective reactions are possible in the presence of multiple C–H bonds (Scheme 1c, Fig. 1b). For thiophene substrates, the selectivity and mechanistic studies support a carboxylation pathway that proceeds *via* C–H deprotonation by the amorphous CO_3_^2−^, as seen previously with arenes. For more nucleophilic indole substrates, however, carboxylation proceeds *via* electrophilic aromatic substitution, which provides a new pathway for CO_2_ utilization enabled by dispersed carbonate materials.

## Results and discussion

### Acidity calculations

**Fig. 2:**
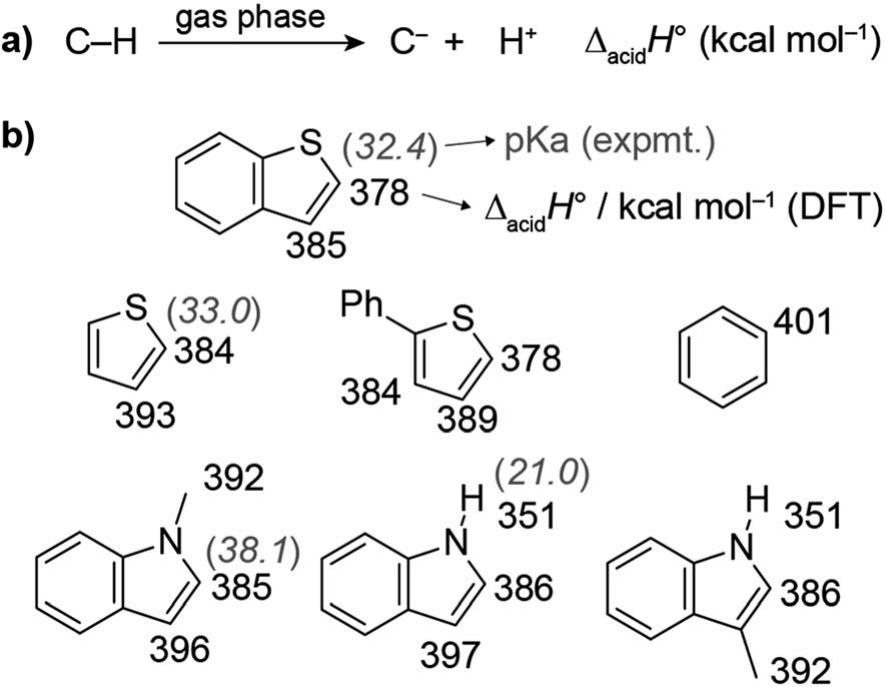
(a) Schematic depiction of gas-phase heterolytic C–H bond dissociation. The standard enthalpy of this reaction is the gas phase acidity. (b) Calculated gas phase acidities and experimental p*K*_a_s of the most acidic C–H bonds in the heteroarene substrates. Benzene is included as a reference point.

Thiophene- and indole-based heterocycles were selected as C–H carboxylation substrates to probe the effects of C–H acidity and π-nucleophilicity. The C–H acidities were evaluated by using density functional theory (DFT) to calculate the standard enthalpy change for heterolytic bond dissociation in the gas phase (Δ_acid_*H*°, also known as the gas phase acidity) (Fig. 2 and S1†).^35,36^ Δ_acid_*H*° provides a way to compare the thermodynamics of deprotonation irrespective of whether the p*K*_a_ can be measured. Benzene, which reacts with dispersed carbonates at ∼400 °C, has a Δ_acid_*H*° of 401 kcal mol^−1^; its p*K*_a_ is too large to be measured but has been estimated to be >43.^36^ The most acidic C–H bonds in each heterocycle were found to be more acidic (lower Δ_acid_*H*°) than benzene by 15–23 kcal mol^−1^, while the separation between the two most acidic C–H bonds in each substrate was 6–11 kcal mol^−1^. For comparison, the experimental p*K*_a_ values of benzothiophene (C2), thiophene (C2), and 1-methylindole (C2) are 32, 33, and 38 according to measurements performed in THF.^36,37^ Additional DFT calculations to determine solution state p*K*_a_ values showed good agreement to these experimental values (Fig. S2†).

### Carbonate-promoted C–H carboxylation reactions

C–H carboxylation reactions were performed in a sealed vessel containing M_2_CO_3_ dispersed on TiO_2_ (M_2_CO_3_/TiO_2_, M^+^ = Cs^+^, K^+^, Na^+^), heterocycle substrate, and CO_2_ (see ESI† for detailed experimental procedures). In most cases, the substrate was placed within a glass culture tube in the reactor to ensure that only volatilized substrate would be able to react with the M_2_CO_3_/TiO_2_ material (Fig. S3†). The products were isolated by aqueous extraction from the TiO_2_ support and quantified by ^1^H NMR (Fig. S4–S9†). In all cases, control experiments using M_2_CO_3_ without the TiO_2_ support showed no reactivity, whereas M_2_CO_3_/TiO_2_ promoted C–H carboxylation in varying degrees depending on the identity of M^+^. Additional control experiments showed minimal reactivity with the mesoporous TiO_2_ support alone.

**Fig. 3:**
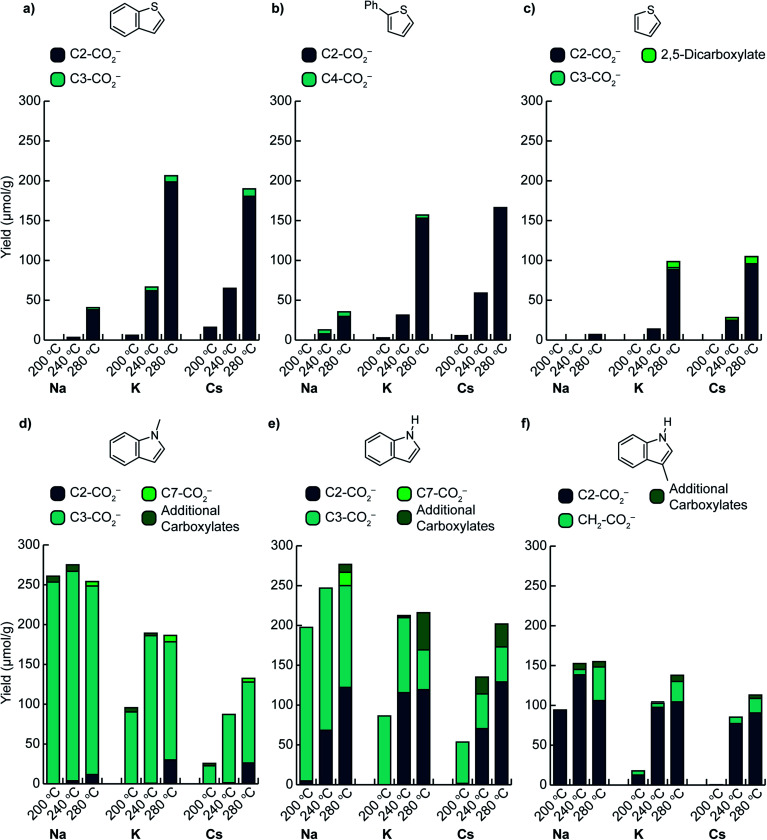
Summary of C–H carboxylation for heteroarenes using M_2_CO_3_/TiO_2_ and CO_2_ at various temperatures ((a) benzothiophene, (b) 2-phenylthiophene, (c) thiophene, (d) 1-methylindole, (e) indole, (f) 3-methylindole). Reactions were performed using glass culture tubes to separate un-vaporized organics from the M_2_CO_3_/TiO_2_ materials. Conditions: 250 mg M_2_CO_3_/TiO_2_, 1.5 mmol heterocycle, 2.5 bar CO_2_ at 298 K, 3 h reaction time.

We first assessed the temperature dependence of C–H carboxylation under a common set of conditions using 1.5 mmol substrate, a CO_2_ loading corresponding to 4–5 bar at the reaction temperature, and a reaction time of 3 h (Fig. 3 and S10†). The relatively low substrate loading corresponded to a maximum pressure of ∼2.5 bar at the highest temperature evaluated (320 °C). Thus, the overall pressure of the reactor at temperature was <8 bar for all of the reactions in this temperature screen. For benzothiophene (Fig. 3a), the onset of carboxylation reactivity was observed at 200 °C. Optimal results were seen at 280 °C, where 190 μmol of benzothiophene carboxylation product was obtained per gram of TiO_2_ (190 μmol g^−1^ TiO_2_) with a 20 : 1 ratio of 2-carboxylate to 3-carboxylate isomers for Cs_2_CO_3_/TiO_2_. Using K_2_CO_3_/TiO_2_, 207 μmol g^−1^ TiO_2_ of benzothiophene carboxylate product was obtained with a 25 : 1 product ratio (Fig. 3a). While both the yield and selectivity declined at higher temperatures, the carboxylation selectivity followed the C–H acidities, consistent with a mechanism gated by C–H deprotonation (see below). In contrast to Cs^+^ and K^+^, much lower reactivity was observed with Na_2_CO_3_/TiO_2_, suggesting that this material is a weaker base in gas–solid reactions.

Comparison of benzothiophene carboxylation with our previous results for benzene carboxylation further highlights the effect of C–H acidity on carboxylation. Whereas >200 μmol g^−1^ TiO_2_ of carboxylate products were obtained for benzothiophene at 280 °C and <8 bar total pressure, the maximum yields for benzene carboxylation using the same M_2_CO_3_/TiO_2_ materials were ∼100 μmol g^−1^ TiO_2_ at 420–440 °C and ∼30 bar total pressure. Thus, reducing the C–H bond acidity (Δ_acid_*H*°) by 23 kcal mol^−1^ enables higher yielding carboxylation reactions under substantially milder conditions (100 °C lower temperature, 1/3 the total pressure). Furthermore, the benzothiophene results also demonstrate that a 7 kcal mol^−1^ separation in C–H acidity (C2 *vs.* C3 position) is sufficient for selective C–H carboxylation.

Because of its high boiling point (221 °C), the vapor pressure of benzothiophene is expected to reach its saturation pressure at *T* ≤ 240 °C under the conditions used for the data in Fig. 3a (see Table S1† for saturation vapor pressures calculated using the Clausius–Clapeyron equation). As a result, the vapor pressure of benzothiophene varies by ∼5× over the 200–320 °C range examined. To deconvolute temperature dependence from substrate pressure dependence, a series of carboxylation reactions were performed at 280 °C for 3 h using different amounts of benzothiophene corresponding to calculated pressures ranging from 0.5 bar to 3.5 bar, which is approximately the saturation pressure at 280 °C. The total benzothiophene carboxylate yield showed a modest variation from 150 μmol g^−1^ TiO_2_ to 210 μmol g^−1^ TiO_2_ over this range (Fig. S17†). Thus, the temperature dependence of the benzothiophene carboxylation yield in Fig. 3a is primarily a result of the temperature effect on the rate constant.

Phenylthiophene reacted in a very similar manner to benzothiophene. The onset of carboxylation was observed at 200 °C with very high selectivity for the 5-phenylthiophene-2-carboxylate isomer (derived from the most acidic C–H bond) observed up to 280 °C. Comparable yields were observed for Cs_2_CO_3_/TiO_2_ and K_2_CO_3_/TiO_2_, while substantially lower yields were seen for Na_2_CO_3_/TiO_2_ (Fig. 3b). The carboxylate yield varied by ∼50% over a 7-fold variation in phenylthiophene pressure (0.5–3.5 bar) at 320 °C (Fig. S17†). The similarity in the temperature- and pressure-dependent yields for both benzothiophene and phenylthiophene is reflected in their nearly identical Δ_acid_*H*° values for their two most acidic C–H bonds, suggesting that the same mechanism is operative for both substrates. Notably, although separating the substrate with a culture tube in the reactor ensures that it can only interact with the M_2_CO_3_*via* the gas phase, the carboxylation reactions with low-volatility substrates like benzothiophene and phenylthiophene proceed in comparable or better yield when the two are combined directly (Fig. S11†).

In contrast to the heterocycle pressure dependence, increasing CO_2_ pressures were found to significantly inhibit C–H carboxylation for both substrates (Fig. S18†). The CO_2_ pressure dependence was evaluated for benzothiophene and phenylthiophene at 280 °C and 320 °C, respectively. Interestingly, inspection of the culture tubes for both substrates post-reaction revealed increasing amounts of un-vaporized heterocycle with increasing CO_2_ partial pressure (Fig. S18†). While the calculated saturation pressures indicate that all of the 1.5 mmol of substrate should be vaporized at these temperatures, this observation suggests that CO_2_ dissolves in the substrate upon melting and lowers its vapor pressure substantially. An additional contributing factor may be that higher CO_2_ pressure results in the formation of polycarbonate species (*e.g.* C_2_O_5_^2−^) on the M_2_CO_3_/TiO_2_ material, which are weaker bases than CO_3_^2−^, thereby reducing the rate of C–H deprotonation.

C–H carboxylation was also possible with thiophene itself. In the temperature screen performed with 1.5 mmol substrate (Fig. 3c), all of the thiophene is expected to be volatilized over the 200–320 °C range because of its relatively low boiling point (84 °C). The corresponding thiophene pressures range from 2–2.5 bar. In contrast to benzothiophene and phenylthiophene, no thiophene carboxylates were observed at 200 °C, which is consistent with the 6 kcal mol^−1^ higher Δ_acid_*H*° for its C(2)–H bond (Fig. 2). The formation of thiophene-2-carboxylate was observed beginning at 240 °C, with optimal results at 280 °C, where 96 μmol g^−1^ TiO_2_ was formed along with 9 μmol g^−1^ TiO_2_ thiophene-2,5-dicarboxylate when using Cs_2_CO_3_/TiO_2_. Comparable yields were obtained with K_2_CO_3_/TiO_2_, while Na_2_CO_3_/TiO_2_ was much less effective. The observation of thiophene-2,5-dicarboxylate indicates that the initially formed monocarboxylate product undergoes a second C–H carboxylation on the support. In addition to the thiophene carboxylates, ∼25 μmol g^−1^ TiO_2_ of propionate was produced across the temperature range of 240–320 °C (Table S12†). This product arises from an unknown decomposition pathway starting from thiophene or a thiophene carboxylate. The yield of thiophene carboxylates was improved by increasing the thiophene pressure to 5 bar, with a comparable proportion of propionate byproduct (Fig. S17†). In contrast to benzothiophene and phenylthiophene, essentially no CO_2_ pressure dependence was observed for thiophene at 280 °C. Given the much higher volatility of thiophene, CO_2_ has a negligible effect on its vapor pressure at this temperature.

We next investigated the effects of increasing the nucleophilicity of the heterocycle by switching from thiophene to indole substrates.^38^ To avoid the complication of an acidic N–H bond, we first evaluated 1-methylindole. The most acidic C–H position of this substrate is C(2)–H, whose Δ_acid_*H*° (384 kcal mol^−1^) is very close to the C(2)–H bond of thiophene (Fig. 2). The most nucleophilic position, however, is C3,^39^ which has a much less acidic C–H bond (Δ_acid_*H*° of C(3)–H is 11 kcal mol^−1^ higher than C(2)–H). Surprisingly, C–H carboxylation occurred readily at 200 °C with a strong preference for the C3 position (Fig. 3d). Moreover, the yield *increased* substantially as the alkali cation size was decreased, resulting in the highest yields for reactions using Na_2_CO_3_/TiO_2_. Optimal results were obtained using Na_2_CO_3_/TiO_2_ at 200 °C, with a yield of 250 μmol g^−1^ TiO_2_ for the C3-carboxylate (Fig. 3d and S8†). At higher temperatures (*T* > 240 °C) the C2-carboxylate was observed as an additional minor product. The selective formation of the C3 carboxylate is consistent with an EAS mechanism in which C–C bond formation precedes C–H deprotonation. Further support was found in the kinetic isotope effect for C–H carboxylation and DFT calculations (see below). Previously reported methods have achieved selective C3 carboxylation of 1-methylindole with CO_2_, but have required the use of stoichiometric organoaluminum reagents.^25,40,41^ Na_2_CO_3_/TiO_2_ provides a benign and much less resource-intensive alternative.

Selective C3 carboxylation was also observed with indole at 200 °C using M_2_CO_3_/TiO_2_ (Fig. 3e). The M_2_CO_3_ dependence followed the same trend as for 1-methylindole, with optimal results obtained using Na_2_CO_3_/TiO_2_. Because the N–H functionality on indole is much more acidic than the C–H bonds (≥35 kcal mol^−1^ difference in Δ_acid_*H*°), it is likely that indole is rapidly deprotonated by M_2_CO_3_/TiO_2_ to form indolide, which can react reversibly with CO_2_ to form indole-1-carboxylate (N–CO_2_^−^). Given the very low acidity of the C–H bond at C3 (Δ_acid_*H*° = 397 kcal mol^−1^, Fig. 2), the selectivity for C3 carboxylation at 200 °C is consistent with an EAS mechanism in which deprotonated indole is the reactive nucleophile.^39^ Beyond 200 °C, however, the reaction yielded a mixture of C3, C2, and C7 carboxylation products. At 280 °C, C2 carboxylation accounted for 44–64% of the total carboxylation products depending on the choice of M_2_CO_3_. Substitution at C2 is commonly seen alongside C3 in solution-phase EAS reactions with indole.^42^ Both methylindole and indole showed very similar pressure dependences on both heterocycle and CO_2_ partial pressure (Fig. S17 and S18†).

Finally, 3-methylindole (skatole) was evaluated to assess the effects of blocking carboxylation at C3. Carboxylation was observed at C2 with a similar temperature dependence as seen for C2 carboxylation of indole (Fig. 3f). Using Na_2_CO_3_/TiO_2_, 94 μmol g^−1^ TiO_2_ of the C2-carboxylate was obtained at 200 °C. Increasing the temperature to 240 °C boosted the yield to 138 μmol g^−1^ TiO_2_, although minor amounts of additional carboxylates were observed at this temperature, including the product of methyl carboxylation. To our knowledge, C2 carboxylation of 3-methylindole with CO_2_ has not previously been achieved.

### Kinetic isotope effects and DFT calculations to probe C–H carboxylation mechanism

**Fig. 4:**
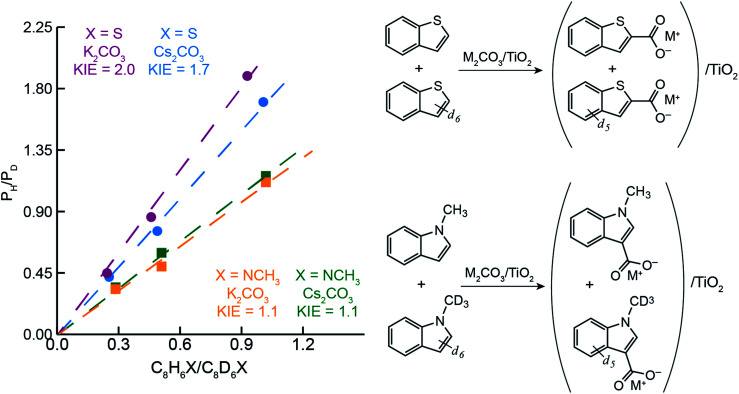
Ratio of protiated to deuterated products obtained from CO_3_^2−^-promoted C–H carboxylation *vs.* the ratio of C_8_H_6_X to C_8_D_6_X (where X = S, NCH_3_, or NCD_3_) at 260 °C and 0.5 h for benzothiophene (top-right) and 200 °C and 1.5 h for methylindole (bottom-right).

**Scheme 2:**
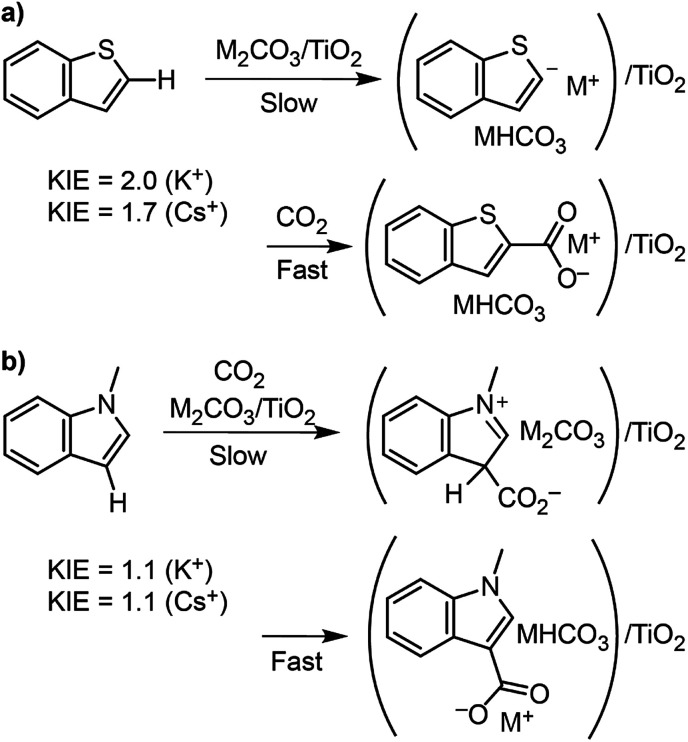
Proposed C–H carboxylation mechanisms for (a) benzothiophene and (b) 1-methylindole. Note that MHCO_3_ is shown for simplicity, but under the reaction conditions it is thermally unstable and will convert to 0.5 equiv. M_2_CO_3_, H_2_O, and CO_2_.

To better understand the differences in thiophene- *vs.* indole-based heteroarene C–H carboxylation, kinetic isotope effects (KIEs) were measured using intermolecular competition experiments.^43^ C–H carboxylation reactions were performed for 1-methylindole (200 °C, 1.5 h) and benzothiophene (260 °C, 0.5 h) using various ratios of protiated and deuterated substrate (Fig. 4).^34^ KIE values of 2.0 and 1.7 were observed for C2 carboxylation of benzothiophene using K_2_CO_3_/TiO_2_ and Cs_2_CO_3_/TiO_2_, respectively. These values are consistent with a mechanism in which C–H deprotonation is slow and the resulting carbanion reacts rapidly with CO_2_ (Scheme 2) and does not support an EAS mechanism. In addition, previous studies of benzothiophene substitution with strong electrophiles have shown selective substitution at C3, indicating that this is the preferred position for EAS reactivity.^39,44,45^ The KIE values for benzothiophene are similar to what we have previously observed for benzene C–H carboxylation using the same M_2_CO_3_/TiO_2_ materials,^34^ as well as solid base-catalyzed reactions that feature rate-determining deprotonation.^46^

In contrast to benzothiophene, a KIE value of 1.1 was observed for C3 carboxylation of 1-methylindole, which is within NMR quantification error of 1.0. The disparity in KIE values for these two substrates indicates distinct mechanisms for their C–H carboxylation reactivity. The lack of a KIE for 1-methylindole is consistent with an EAS mechanism at 200 °C in which attack of the π system on CO_2_ precedes C–H deprotonation (Scheme 2). To our knowledge, an EAS reaction between CO_2_ and a neutral substrate has not previously been reported. DFT calculations were performed to assess the feasibility of such a pathway with 1-methylindole. Calculations performed using either vacuum or low dielectric solvents (*ε* < 9) failed to identify a transition state or putative EAS intermediate, suggesting that a gas-phase reaction between 1-methylindole and CO_2_ is unlikely. With a higher dielectric (*ε* > 20), however, an EAS transition state was identified that is ∼30 kcal mol^−1^ higher in energy than the substrates (Fig. S19†). Interestingly, the zwitterionic intermediate resulting from CO_2_ addition was very close in energy to the transition state, indicating that the reverse reaction is extremely rapid. Together, the KIE and DFT results suggest that the carboxylation of methylindole takes place *via* an EAS mechanism with substrate that is adsorbed onto the M_2_CO_3_/TiO_2_ material. The amorphous carbonate provides a dielectric to stabilize the transition state for CO_2_ addition and a proximal base that can immediately deprotonate the putative zwitterionic intermediate. The higher yield for Na_2_CO_3_/TiO_2_ may reflect a stronger adsorption of 1-methylindole because of the higher charge density for Na^+^. Further studies incorporating atomistic modeling of the amorphous carbonate surface are needed to assess this pathway more thoroughly. Nonetheless, the DFT results indicate that an EAS-like mechanism is possible.

### Carboxylate esterification and M_2_CO_3_/TiO_2_ regeneration

**Fig. 5:**
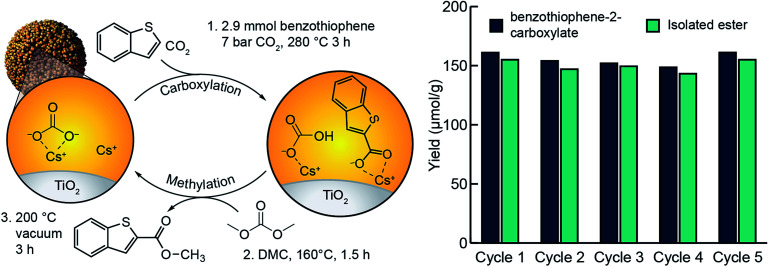
Cycling steps and yields for benzothiophene esterification using Cs_2_CO_3_/TiO_2_. Reaction conditions: carboxylation at 280 °C for 3 h, esterification at 160 °C for 1.5 h, followed by a regeneration cycle at 200 °C for 3 h under reduced pressure.

In our previous study of arene C–H carboxylation, we showed that arene carboxylates could be isolated as methyl esters with concomitant regeneration of the M_2_CO_3_/TiO_2_ material by subjecting the carboxylation product to flowing CO_2_ and methanol at elevated temperatures.^34^ The same procedure was unsuccessful for isolating heteroarene carboxylate esters because their high boiling points (>300 °C) necessitated temperatures that led to decomposition under the reaction conditions. The use of flowing CO_2_ and dimethyl carbonate enabled isolation of methyl esters, but the yields were <50% (Fig. S20†). Instead, it was found that we could isolate the ester at near quantitative yields by heating the supported heteroarene carboxylate ((RCOOM)/TiO_2_) in neat dimethyl carbonate at 160 °C within a stainless-steel batch reactor (Fig. S21†). Subsequent heating of the support material under vacuum resulted in regeneration of M_2_CO_3_/TiO_2_. After establishing optimal carboxylation and methylation conditions, we assessed the ability of M_2_CO_3_/TiO_2_ to catalyze a closed heteroarene esterification cycle over multiple iterations (Fig. 5). When a single sample of Cs_2_CO_3_/TiO_2_ was used for 5 cycles, methyl benzothiophene-2-carboxylate was isolated as the only detectable product by NMR (Fig. S16†) from each cycle with an average yield of 150 μmol g^−1^ TiO_2_. In each cycle following the methylation step, an aliquot of the support (∼50 mg) was analyzed by aqueous extraction and ^1^H NMR to detect unreacted, supported carboxylate. In all cases, no supported carboxylates were observed, indicating complete methylation. Over the five cycles, no indication of catalyst degradation was observed (Fig. 5). These results support previous observations of the ability for dispersed carbonates to catalyze a closed esterification cycle,^34^ and extend the substrate scope to include heteroarenes.

## Conclusion

Conventional solution-phase methods for C–H carboxylation of aromatic substrates with low C–H acidity have relied on the use of highly reactive and resource-intensive organic bases. Our results show that CO_3_^2−^ can serve as a benign, regenerable base for C–H carboxylation *via* a gas–solid reaction utilizing a dispersed, amorphous carbonate material. Compared to reactions with benzene and other arenes using the same M_2_CO_3_/TiO_2_ materials, the heteroarene carboxylations investigated here reach higher yields (up to 250 μmol g^−1^ TiO_2_) under substantially milder conditions (200 °C lower temperature, 1/3 the total pressure). Thiophene-based heterocycles react preferentially at the most acidic C–H bond. The temperature-dependent selectivity and KIE measured for benzothiophene are consistent with a mechanism in which C–H deprotonation is followed by C–C bond formation. In contrast, indole-based heterocycles react preferentially at the most nucleophilic position (C3). DFT calculations and the absence of a significant KIE support an EAS mechanism for the carboxylation of 1-methylindole, which nonetheless requires dispersed carbonate. The combination of CO_3_^2−^-promoted C–H carboxylation and methylation with dimethyl carbonate provides a two-step cycle to convert aromatic heteroarenes into methyl esters with regeneration of M_2_CO_3_/TiO_2_. Ongoing work seeks to improve the efficiency of this cycle by using alternative supports to increase the loading of reactive carbonate and access reactivity at lower temperatures.

## Conflicts of interest

The authors declare no competing financial interests.

## Supplementary Material

SC-011-D0SC04548A-s001

## References

[cit1] Tomassi I. (2017). Direct Carboxylation of C(sp^3^)–H and C(sp^2^)–H Bonds with CO_2_ by Transition-Metal-Catalyzed and Base-Mediated Reactions. Catalysts.

[cit2] Luo J., Larrosa I. (2017). C–H Carboxylation of Aromatic Compounds through CO_2_ Fixation. ChemSusChem.

[cit3] Hong J., Li M., Zhang J., Sun B., Mo F. (2019). C–H Bond Carboxylation with Carbon Dioxide. ChemSusChem.

[cit4] Vechorkin O., Hirt N., Hu X. (2010). Carbon Dioxide as the C1 Source for Direct C–H Functionalization of Aromatic Heterocycles. Org. Lett..

[cit5] Pieber B., Glasnov T., Kappe C. O. (2014). Flash carboxylation: fast lithiation–carboxylation sequence at room temperature in continuous flow. RSC Adv..

[cit6] Yoo W.-J., Nguyen T. V. Q., Capdevila M. G., Kobayashi S. (2015). Lithium *tert*-Butoxide-Mediated Carboxylation Reactions of Unprotected Indoles and Pyrroles with Carbon Dioxide. Heterocycles.

[cit7] Fenner S., Ackermann L. (2016). C–H carboxylation of heteroarenes with ambient CO_2_. Green Chem..

[cit8] Luo J., Preciado S., Xie P., Larrosa I. (2016). Carboxylation of Phenols with CO_2_ at Atmospheric Pressure. Chem.–Eur. J..

[cit9] Shigeno M., Hanasaka K., Sasaki K., Nozawa-Kumada K., Kondo Y. (2019). Direct Carboxylation of Electron-Rich Heteroarenes Promoted by LiO-*t*Bu with CsF and [18]Crown-6. Chem.–Eur. J..

[cit10] Seo H., Katcher M. H., Jamison T. F. (2017). Photoredox activation of carbon dioxide for amino acid synthesis in continuous flow. Nat. Chem..

[cit11] Ishida N., Masuda Y., Imamura Y., Yamazaki K., Murakami M. (2019). Carboxylation of Benzylic and Aliphatic C–H Bonds with CO_2_ Induced by Light/Ketone/Nickel. J. Am. Chem. Soc..

[cit12] Meng Q.-Y., Schirmer T. E., Berger A. L., Donabauer K., König B. (2019). Photocarboxylation of Benzylic C–H Bonds. J. Am. Chem. Soc..

[cit13] Seo H., Nguyen L. V., Jamison T. F. (2019). Using Carbon Dioxide as a Building Block in Continuous Flow Synthesis. Adv. Synth. Catal..

[cit14] Yeung C. S. (2019). Photoredox Catalysis as a Strategy for CO_2_ Incorporation: Direct Access to Carboxylic Acids from a Renewable Feedstock. Angew. Chem., Int. Ed..

[cit15] Carbon Dioxide as Chemical Feedstock, ed. M. Aresta, Wiley-VCH, Weinheim, 2010

[cit16] Börjesson M., Moragas T., Gallego D., Martin R. (2016). Metal-Catalyzed Carboxylation of Organic (Pseudo)halides with CO_2_. ACS Catal..

[cit17] Gooßen L. J., Rodríguez N., Gooßen K. (2008). Carboxylic Acids as Substrates in Homogeneous Catalysis. Angew. Chem., Int. Ed..

[cit18] Kumar D., Maruthi Kumar N., Chang K.-H., Shah K. (2010). Synthesis and anticancer activity of 5-(3-indolyl)-1,3,4-thiadiazoles. Eur. J. Med. Chem..

[cit19] Maag H., Prodrugs of Carboxylic Acids, in Prodrugs: Challenges and Rewards Part 1, ed. V. J. Stella, R. T. Borchardt, M. J. Hageman, R. Oliyai, H. Maag and J. W. Tilley, Springer New York, New York, NY, 2007, pp. 703–729

[cit20] Boullard O., Leblanc H. and Besson B., Salicylic Acid, Ullmann's Encyclopedia of Industrial Chemistry, 2000

[cit21] Yoo W.-J., Capdevila M. G., Du X., Kobayashi S. (2012). Base-Mediated Carboxylation of Unprotected Indole Derivatives with Carbon Dioxide. Org. Lett..

[cit22] Pei C., Zong J., Han S., Li B., Wang B. (2020). Ni-Catalyzed Direct Carboxylation of an Unactivated C–H Bond with CO_2_. Org. Lett..

[cit23] Mizuno H., Takaya J., Iwasawa N. (2011). Rhodium(i)-Catalyzed Direct Carboxylation of Arenes with CO_2_ via Chelation-Assisted C–H Bond Activation. J. Am. Chem. Soc..

[cit24] Fu L., Li S., Cai Z., Ding Y., Guo X.-Q., Zhou L.-P., Yuan D., Sun Q.-F., Li G. (2018). Ligand-enabled site-selectivity in a versatile rhodium(ii)-catalysed aryl C–H carboxylation with CO_2_. Nat. Catal..

[cit25] Suga T., Mizuno H., Takaya J., Iwasawa N. (2014). Direct carboxylation of simple arenes with CO_2_ through a rhodium-catalyzed C–H bond activation. Chem. Commun..

[cit26] Schlosser M., Jung H. C., Takagishi S. (1990). Selective mono- or dimetalation of arenes by means of superbasic reagents. Tetrahedron.

[cit27] Olah G. A., Török B., Joschek J. P., Bucsi I., Esteves P. M., Rasul G., Surya Prakash G. K. (2002). Efficient Chemoselective Carboxylation of Aromatics to Arylcarboxylic Acids with a Superelectrophilically Activated Carbon Dioxide—Al_2_Cl_6_/Al System. J. Am. Chem. Soc..

[cit28] Schmalzbauer M., Svejstrup T. D., Fricke F., Brandt P., Johansson M. J., Bergonzini G., König B. (2020). Redox-Neutral Photocatalytic C–H Carboxylation of Arenes and Styrenes with CO_2_. Chem.

[cit29] Banerjee A., Dick G. R., Yoshino T., Kanan M. W. (2016). Carbon dioxide utilization via carbonate-promoted C–H carboxylation. Nature.

[cit30] Dick G. R., Frankhouser A. D., Banerjee A., Kanan M. W. (2017). A scalable carboxylation route to furan-2,5-dicarboxylic acid. Green Chem..

[cit31] Banerjee A., Kanan M. W. (2018). Carbonate-Promoted Hydrogenation of Carbon Dioxide to Multicarbon Carboxylates. ACS Cent. Sci..

[cit32] Lankenau A. W., Kanan M. W. (2020). Polyamide monomers via carbonate-promoted C–H carboxylation of furfurylamine. Chem. Sci..

[cit33] Frankhouser A. D., Kanan M. W. (2020). Phase Behavior That Enables Solvent-Free Carbonate-Promoted Furoate Carboxylation. J. Phys. Chem. Lett..

[cit34] Xiao D. J., Chant E. D., Frankhouser A. D., Chen Y., Yau A., Washton N. M., Kanan M. W. (2019). A closed cycle for esterifying aromatic hydrocarbons with CO_2_ and alcohol. Nat. Chem..

[cit35] Anslyn E. V. and Dougherty D. A., Modern Physical Organic Chemistry, University Science Books, 2006

[cit36] Shen K., Fu Y., Li J.-N., Liu L., Guo Q.-X. (2007). What are the p*K*_a_ values of C–H bonds in aromatic heterocyclic compounds in DMSO?. Tetrahedron.

[cit37] Fraser R. R., Mansour T. S., Savard S. (1985). Acidity measurements in THF. V. Heteroaromatic compounds containing 5-membered rings. Can. J. Chem..

[cit38] Mayr H., Kempf B., Ofial A. R. (2003). π-Nucleophilicity in Carbon–Carbon Bond-Forming Reactions. Acc. Chem. Res..

[cit39] Eicher T., Hauptmann S. and Speicher A., Five-Membered Heterocycles: Sections 5.1–5.21, The Chemistry of Heterocycles, 2003, pp. 52–121

[cit40] Nemoto K., Tanaka S., Konno M., Onozawa S., Chiba M., Tanaka Y., Sasaki Y., Okubo R., Hattori T. (2016). Me_2_AlCl-mediated carboxylation, ethoxycarbonylation, and carbamoylation of indoles. Tetrahedron.

[cit41] Tanaka S., Watanabe K., Tanaka Y., Hattori T. (2016). EtAlCl_2_/2,6-Disubstituted Pyridine-Mediated Carboxylation of Alkenes with Carbon Dioxide. Org. Lett..

[cit42] Westermaier M., Mayr H. (2006). Electrophilic Allylations and Benzylations of Indoles in Neutral Aqueous or Alcoholic Solutions. Org. Lett..

[cit43] Simmons E. M., Hartwig J. F. (2012). On the Interpretation of Deuterium Kinetic Isotope Effects in C–H Bond Functionalizations by Transition-Metal Complexes. Angew. Chem., Int. Ed..

[cit44] Ji Ram V., Sethi A., Nath M. and Pratap R., Chapter 5 – Five-Membered Heterocycles, in The Chemistry of Heterocycles, ed. V. Ji Ram, A. Sethi, M. Nath and R. Pratap, Elsevier, 2019, pp. 149–478

[cit45] Kajorinne J. K., Steers J. C. M., Merchant M. E., MacKinnon C. D. (2018). Green halogenation reactions for (hetero)aromatic ring systems in alcohol, water, or no solvent. Can. J. Chem..

[cit46] Ono Y. and Hattori H., Characterization of Solid Base Catalysts, in Solid Base Catalysis, ed. Y. Ono and H. Hattori, Springer Berlin Heidelberg, Berlin, Heidelberg, 2011, pp. 11–68

