# Supplementary material for: Carbonate-promoted C–H carboxylation of electron-rich heteroarenes
Source: Chem Sci. 2020 Oct 5;11(43):11936–44. doi: 10.1039/d0sc04548a (PMC8162799; doi:10.1039/d0sc04548a)
Supplement: SC-011-D0SC04548A-s001 [file SC-011-D0SC04548A-s001.pdf]

Electronic Supplementary Material (ESI) for Chemical Science.
This journal is © The Royal Society of Chemistry 2020

Supplementary Information

Carbonate-Promoted C–H Carboxylation of Electron-Rich Heteroarenes

Tyler M. Porter and Matthew W. Kanan*

Department of Chemistry, Stanford University, Stanford, California, USA

Table of Contents.

1. General procedures S2–S4
2. NMR references S5–S6
3. Supplementary figures S7–S27
4. Supplementary tables S28–S42

Density Functional Theory Calculations

Density functional theory (DFT) calculations were performed using the Gaussian 16 software suite.[1] All energies are reported as standard Gibbs free energies (ΔG), with thermal corrections to the Gibbs free energies obtained via frequency calculations that employed the harmonic oscillator, rigid rotor, and ideal gas approximations. All structures have been characterized by frequency calculations as either a minimum (no imaginary modes) or a transition state (one imaginary vibration mode). The transition states (TS) were additionally characterized by means of intrinsic reaction coordinate (IRC) calculations to verify that a particular TS structure connects the correct energy minima.

Geometry optimization and frequency calculations for gas phase acidity and pK_a calculations were performed at the B3LYP/6–311++G(3df,2p) level of theory, with the conductor-like polarizable continuum model (CPCM) to mimic DMSO solvation in pK_a calculations. Following literature precedent,[2] the gas phase acidities and solution state pK_a values were determined for the five heterocycles by utilizing the dissociation and isodesmic reactions represented in **Figs. S1** and **S2**. Transition state searches and thermochemical calculations pertaining to the electrophilic aromatic substitution reaction depicted in **Scheme 2** were performed at the B3LYP/6–31G+(d,p) level of theory with CPCM to mimic benzene, quinoline, 1-propanol, acetonitrile, dmso, and water solvation.

General Carboxylation Procedure

Alkali carbonates dispersed in mesoporous TiO_2 nanoparticles (M_2CO_3/TiO_2) were prepared using our previously reported procedures,[3-4] with carbonate loadings of 0.56 mmol per gram TiO_2 (0.56 mmol g^{-1} TiO_2) for Na_2CO_3/TiO_2, 0.52 mmol g^{-1} TiO_2 for K_2CO_3/TiO_2 and 0.47 mmol g^{-1} TiO_2 for Cs_2CO_3/TiO_2. Prior to a carboxylation reaction, small stainless-steel batch reactors ($V_{total} \approx 27.5$ mL), built from Swagelok single-ended miniature sample cylinders (25 mL), were dried at 150 °C in an oven for a minimum of 3 h, and then cooled to room temperature under vacuum. The batch reactors were then loaded with M_2CO_3/TiO_2 (~250 mg) and an amount of substrate (1.5 mmol) isolated in a glass culture tube (50 x 6 mm, **Fig. S3**). The culture tube was needed to separate any un-vaporized substrate from the M_2CO_3/TiO_2 material. The extent of vaporization for phenylthiophne as a function of temperature can clearly be seen in **Fig. S3**.

After substrate addition, the reactor was sealed, attached to a stainless-steel gas dosing manifold, and degassed by three, freeze-pump-thaw cycles, followed by the addition of ~2.5 bar CO_2 at room temperature. The reactor was then removed from the manifold, wrapped in heat tape and insulation, and subsequently heated to the desired temperature at a rate of 20 °C min^{-1}. After heating for 3 h, the reactor was cooled to room temperature, depressurized, and dismantled. The culture tubes were removed using tweezers while the supported carboxylate products ($RCOOM/TiO_2$, also containing unreacted supported M_2CO_3) could be transferred as a free-flowing powder to a 20 mL scintillation vial. The reactor was subsequently washed with diethyl ether (3 x 2 mL) to remove any unreacted organics and transferred to the same 20 mL vial containing the $RCOOM/TiO_2$ material. The diethyl ether was then separated by centrifugation and subsequently placed under a stream of N_2 at room temperature to isolate unreacted organics. Analysis from representative reactions indicated recovery of 70-90% of the unreacted substrate. The supported carboxylate products were then separated from the TiO_2 support via aqueous extraction (3 x 2 mL, 70 °C) and filtration through a syringe filter (0.2 μm pores, PVDF). The aqueous extract was then heated to 125 °C under a stream of N_2 to remove H_2O. The carboxylate

products were analyzed by ^{1}H NMR in D_2O and quantified using a sodium pivalate internal standard (**Figs. S3–S8**), while the Et_2O extracted organics were analyzed by ^{1}H NMR in CD_3OD.

Carboxylation Procedure without Separating the Substrate

While not required, the use of a glass culture tube ensured that any un-vaporized substrate was separated from the M_2CO_3/TiO_2 base and that carboxylation proceeded by a gas–solid reaction (**Fig. S11**). It was found, however, that for all reactions studied the substrate could be directly mixed with the M_2CO_3/TiO_2 material in the reactor. Much like the culture tube reactions, the direct mixing experiments were performed under a standard set of conditions utilizing the same Swagelok mini-reactors loaded with M_2CO_3/TiO_2 (~250 mg), an excess amount of substrate (2.9 mmol), and ~4.5 bar of CO_2 at room temperature. The excess loading of substrate was found to exceed the saturation vapor pressure for all substrates, except thiophene, when the reaction temperature was below 320 °C (**Table S1**). The presence of a liquid-vapor equilibrium did not impede the carboxylation reaction. In fact, under optimal conditions, the yields for the three thiophene substrates were found to improve in comparison to the culture tube methodology (ΔYield: Benzothiophene = 30 μmol g^{-1} TiO_2, Phenylthiophene = 11 μmol g^{-1} TiO_2, Thiophene = 24 μmol g^{-1} TiO_2) when Cs_2CO_3/TiO_2 was used (**Figs. 3** and **S11**). The indole substrates displayed a similar trend at 240 °C, where the total carboxylate yield for indole and 1-methylindole could be increased by an additional 49 μmol g^{-1} TiO_2 and 35 μmol g^{-1} TiO_2, respectively, when Cs_2CO_3/TiO_2 was used.

Kinetic Isotope Measurements

Benzothiophene-d_6 and indole-d_7 were synthesized following an adapted literature procedure.[5] In brief: A Teflon lined Parr acid digestion vessel (125 mL, Model: 4748) was charged with 2.5 g of substrate (~19 mmol), 431 mg of PtO_2 (1.9 mmol), and 70 g of D_2O. The vessel was then sealed and placed in a temperature-controlled oven set to 240 °C for 6-12 h. After cooling to room temperature, the organics were extracted into chloroform and the Teflon vessel was washed and sonicated for 15 min with an additional 70 mL of chloroform. The combined organic layers were then filtered to remove any residual PtO_2 catalyst, dried over Na_2SO_4, and concentrated under vacuum. For benzothiophene, the obtained brown oil was further purified by sublimation at 100 °C and 10 mTorr to yield a white crystalline powder identified as benzothiophene-d_6 (**Fig. S12**).

The brown powder obtained from the indole-d_7 synthesis was subsequently used as is to synthesize methylindole-d_9 following literature precedent.[6] In brief, a 40 mL scintillation vial was charged with 4.00 g (32.2 mmol) indole-d_7, 2.169 g (38.7 mmol) potassium hydroxide, and 20 mL anhydrous dimethyl sulfoxide. The resulting reaction mixture was stirred at room temperature for 20 min, whereby 2.89 mL (46.4 mmol) iodomethane-d_3 was subsequently added dropwise over the course of 2 min. The vial was then sealed and left to stir for an additional 6 h. After stirring, the organics were extracted into 100 mL of ethyl acetate, and subsequently washed with brine (5 x 30 mL) to remove dimethyl sulfoxide, dried over anhydrous sodium sulfate, and concentrated under reduced pressure to yield a dark brown oil. The methylindole-d_9 was purified by column chromatography (4:1 hexanes:ethyl acetate) and isolated as a clear oil as the first species to elute (**Fig. 2**.

The carboxylation reactions performed to measure kinetic isotope effects were performed following the general procedure described above except that culture tubes were not used to separate the substrates from the M_2CO_3/TiO_2 materials. Experiments were performed using mixtures of proto- and duetero-heterocycle (1:1, 1:2, and 1:4) at 200 °C (1.5 h) for methylindole and 260 °C

(0.5 h) for benzothiophene. Reduced times were used in both cases to prevent H/D scrambling. Following carboxylation, the alkali salts were extracted from the support (3 x 10 mL H_2O, 70 °C), dried to a solid, and subsequently analyzed by ^{1}H and ^{2}H NMR in D_2O using *t*-butanol and DMSO-*d*$_6$ as internal standards respectively (**Figs. S13** and **S14**).

General Esterification Procedure

A single, large scale (~1.25 g M_2CO_3/TiO_2) benzothiophene carboxylation was performed with Cs_2CO_3/TiO_2 at 280 °C. The total amount of supported carboxylate was then quantified by aqueous extraction using ~50 mg of the support and ^{1}H NMR analysis. Esterification reactions were performed by loading the mini-reactor (**Fig. S21**) with ~100 mg of the supported carboxylate and 1 mL (11.9 mmol) of dimethyl carbonate. The reactor was sealed, attached to a gas dosing manifold, and degassed by three, freeze-pump thaw cycles. One bar of N_2 was then added at room temperature and the reactor was removed from the manifold, wrapped in heat tape/insulation, and heated to the specified temperature at a rate of 20 °C min^{-1}. After the reaction, the reactor was cooled to room temperature, depressurized, and dismantled. The reactor was subsequently washed with diethyl ether (3 x 2 mL) to remove excess dimethyl carbonate and any volatilized carboxylate ester. The organic extract was isolated from the TiO_2 support by centrifugation and placed under a stream of N_2 at room temperature to remove Et_2O. The organic extract was analyzed by ^{1}H NMR in CD_3OD and quantified using a *t*-butanol internal standard. The unreacted supported carboxylate salts were separated from the TiO_2 support via aqueous extraction (3 x 2 mL, 70 °C) and filtered through a syringe filter (0.2 μm pores, PVDF). The aqueous extract was then heated to 125 °C under a stream of N_2 to remove H_2O, and the carboxylate products were analyzed by ^{1}H NMR in D_2O and quantified using a sodium pivalate internal standard (**Figs. S15** and **S16**).

General Carboxylation/Esterification Cycling Procedure

The cycling studies were performed in a similar manner whereby a large scale (~1.25 g Cs_2CO_3/TiO_2) benzothiophene carboxylation was performed at 280 °C using 7 bar CO_2 and 2.9 mmol benzothiophene. The total amount of supported carboxylate product was quantified by aqueous extraction using ~50 mg of the support and ^{1}H NMR analysis. The remaining ~1.2 g of supported carboxylate was then added to the mini-reactor (**Fig. S21**) with 2 mL of dimethyl carbonate. The reactor was sealed, attached to a gas dosing manifold, and degassed by three, freeze-pump thaw cycles. One bar of N_2 was then added at room temperature and the reactor was removed from the manifold, wrapped in heat tape/insulation, and heated to 160 °C at a rate of 20 °C min^{-1} for 1.5 h. After the esterification step, the reactor was cooled to room temperature, depressurized, and dismantled. The reactor was subsequently washed with diethyl ether (3 x 2 mL) to remove excess dimethyl carbonate and any volatilized carboxylate ester. The organic extract was isolated from the TiO_2 support by centrifugation and placed under a stream of N_2 at room temperature to remove Et_2O. The remaining post-esterified support was then analyzed for un-reacted carboxylates by aqueous extraction using a small aliquot (~50 mg) of the bulk material. Both organic and aqueous extracts were analyzed by ^{1}H NMR using CD_3OD and D_2O respectively. Products were quantified by using an internal standard of *t*-butanol (organic extract) or sodium pivalate (aqueous extract). After product quantification, the remaining ~1.15 g of support was regenerated by heating to 200 °C for 3 h under reduced pressure. The above procedure was then repeated for four additional cycles.

Product Analysis and NMR References

NMR peaks of the carboxylation products were assigned by comparison to spectra of the Cs^+ salts of the pure compounds obtained independently from the pure carboxylic acid. The resonances for these compounds are provided below for reference. (Note: Depending on the salt/base concentration of the D_2O sample, the ^1H peaks may shift slightly compared to these references.)

Cesium benzothiophene-2-carboxylate
^1H NMR (300 MHz, D_2O) δ 7.93 (m, 2H), 7.83 (s, 1H), 7.46 (m, 2H)

Cesium benzothiophene-3-carboxylate
^1H NMR (300 MHz, D_2O) δ 8.39 (d, 1H, *J* = 7.9 Hz), 8.11 (s, 1H), 7.97 (d, 1H, *J* = 7.9 Hz), 7.50 (t, 1H, *J* = 7.7 Hz), 7.43 (t, 1H, *J* = 7.7 Hz).

Cesium benzothiophene-7-carboxylate
^1H NMR (300 MHz, D_2O) δ 8.02 (d, 1H, *J* = 7.7 Hz), 7.92 (d, 1H, *J* = 7.5 Hz), 7.65 (d, 1H *J* = 5.6 Hz), 7.48 (m, 2H).

Cesium 5-phenylthiophene-2-carboxylate
^1H NMR (300 MHz, D_2O) δ 7.65 (d, 2H, *J* =7.3 Hz), 7.46 (d, 1H, *J* =3.9 Hz), 7.38 (m, 4H).

Cesium thiophene-2-carboxylate
^1H NMR (300 MHz, D_2O) δ 7.51 (m, 2H), 7.07 (t, 1H, *J* =4.5 Hz).

Cesium thiophene-3-carboxylate
^1H NMR (300 MHz, D_2O) δ 7.90 (s, 1H), 7.40 (m, 2H).

Cesium thiophene-2,5-dicarboxylate
^1H NMR (300 MHz, D_2O) δ 7.43 (s, 2H).

Cesium propionate
^1H NMR (300 MHz, D_2O) δ 2.09 (q, 2H, *J* = 7.6 Hz), 0.96 (t, 3H, *J* = 7.7 Hz).

Cesium indole-2-carboxylate
^1H NMR (300 MHz, D_2O) δ 7.68 (d, 1H, *J* = 8.4 Hz), 7.48 (d, 1H, *J* = 8.3 Hz), 7.27 (t, 1H, *J* = 7.6 Hz), 7.10 (t, 1H, *J* = 7.6 Hz), 6.96 (s, 1H).

Cesium indole-3-carboxylate
^1H NMR (300 MHz, D_2O) δ 8.04 (m, 1H), 7.70 (s, 1H), 7.49 (m, 1H), 7.20 (m, 2H).

Cesium indole-7-carboxylate
^1H NMR (300 MHz, D_2O) δ 7.82 (d, 1H, *J* = 7.4 Hz), 7.70 (d, 1H, *J* = 7.4 Hz), 7.44 (d, 1H, *J* = 3.2 Hz), 7.17 (t, 1H, *J* = 7.8 Hz), 6.62 (d, 1H, *J* = 3.2 Hz).

Cesium 1-methylindole-2-carboxylate

^{1}H NMR (300 MHz, D_2O) δ 7.67 (d, 1H, J = 8.0 Hz), 7.48 (d, 1H, J = 8.4 Hz), 7.33 (t, 1H, J =7.3 Hz), 7.14 (t, 1H, J = 7.5 Hz), 6.97 (s, 1H), 3.91 (s, 3H).

Cesium 1-methylindole-3-carboxylate

^{1}H NMR (300 MHz, D_2O) δ 8.04 (d, 1H, J = 7.3 Hz), 7.67 (s, 1H), 7.42 (d, 1H, J = 7.5 Hz), 7.24 (m, 2H), 3.373 (s, 3H).

Cesium 3-methyl-1H-indole-2-carboxylate

^{1}H NMR (400 MHz, D_2O) δ 7.68 (d, 1H, J = 8.13 Hz), 7.44 (d, 1H, J = 8.13 Hz), 7.28 (t, 1H, J = 7.62 Hz), 7.12 (t, 1H, J = 7.62 Hz), 2.45 (s, 3H).

Methyl benzothiophene-2-carboxylate

^{1}H NMR (400 MHz, MeOD) δ 8.08 (s, 1H), 7.92 (t, 2H, J = 7.3 Hz), 7.44 (m, 2H), 3.92 (s, 3H).

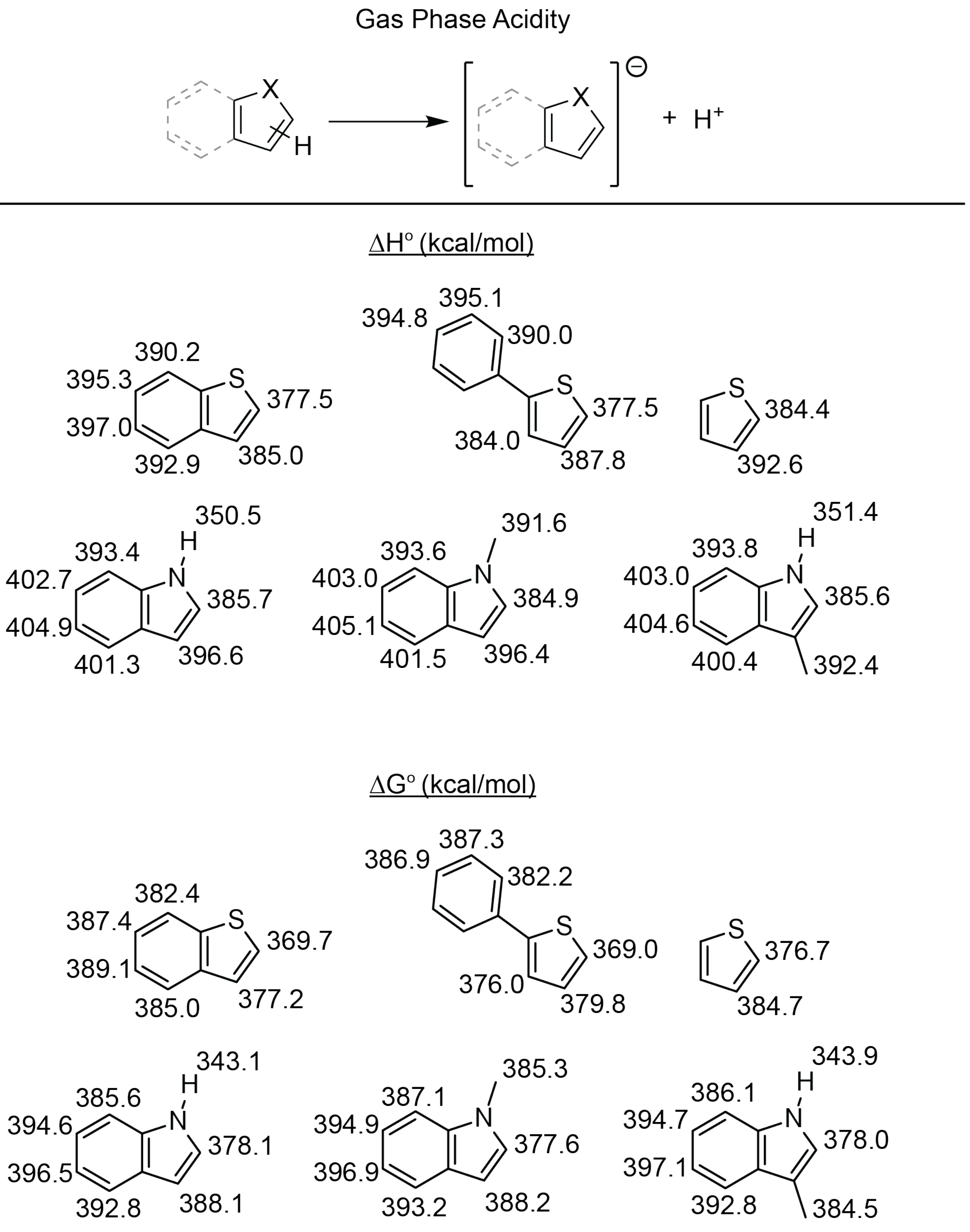

Figure S1. DFT computed gas phase acidities for the five aromatic heterocycles.

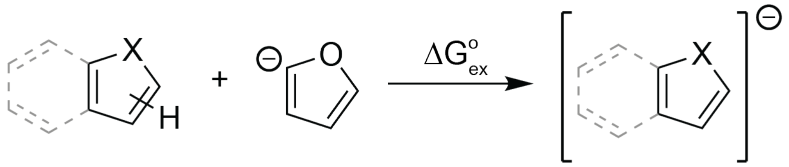

$$pK_a(Het) = 35.0 + \frac{\Delta G^{o}_{ex}}{2.303(RT)}$$

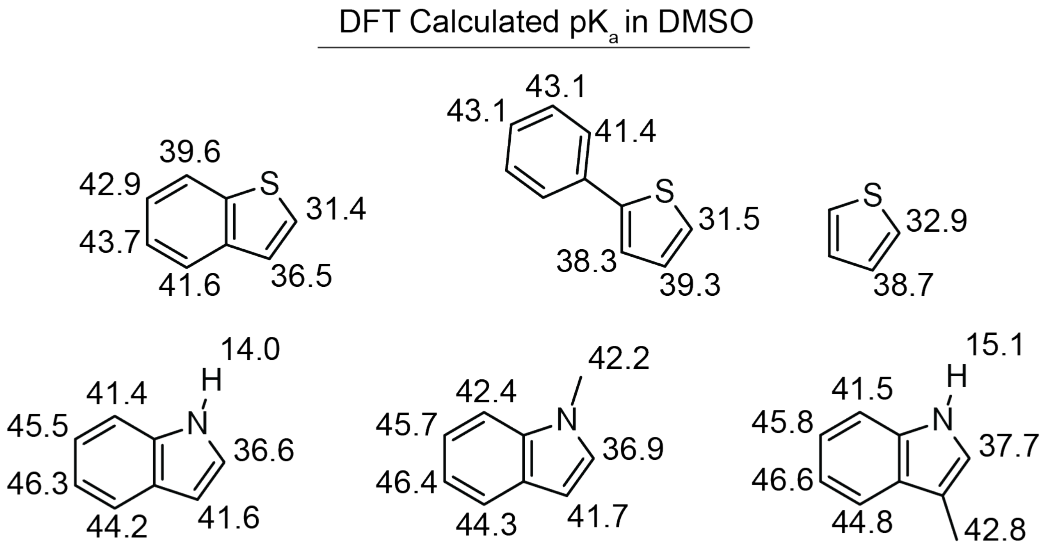

Figure S2. DFT calculated pK_a values for the five heteroarene substrates in DMSO solution. pK_a values are determined using the free energy change of the isodesmic reaction between furan deprotonated at the C2 position and the heteroarene of interest (top). The value of 35.0 is the experimentally determined pK_a of the C(2)–H for furan.[2]

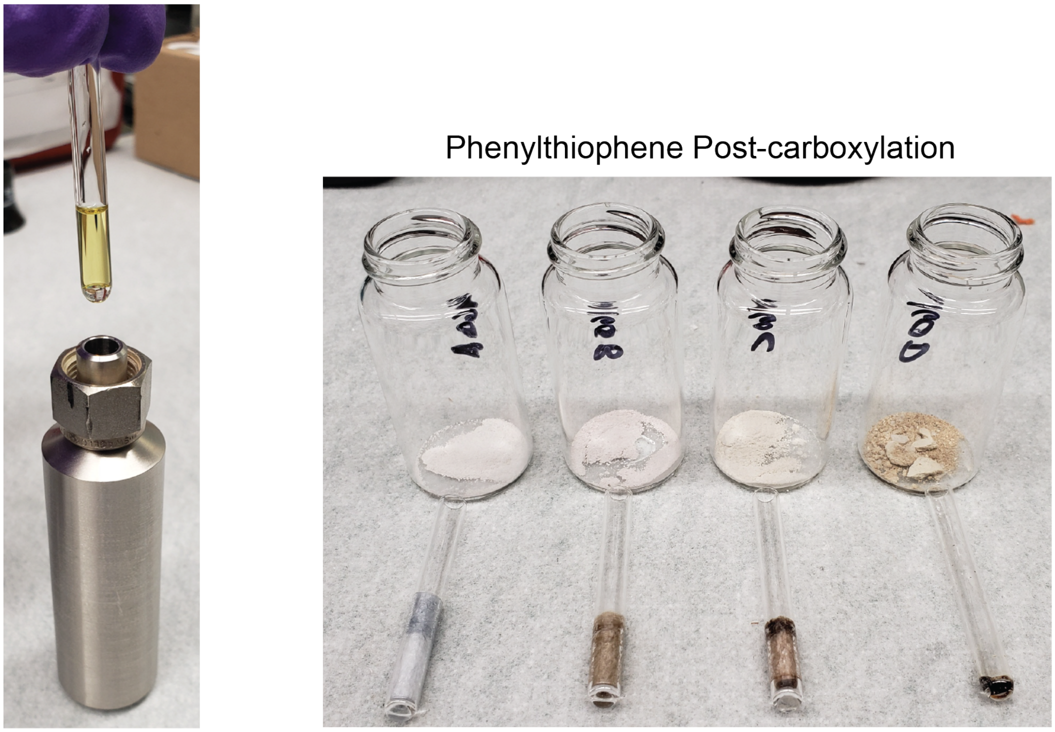

Figure S3. Carboxylation mini-reactor loaded with Cs_2CO_3/TiO_2 and the glass culture tube containing 1-methylindole before a carboxylation reaction (left). Post-carboxylation workup of a phenylthiophene carboxylation reaction with 250 mg Cs_2CO_3/TiO_2 and 239 mg (1.5 mmol) phenylthiophene. The scintillation vials contain the TiO_2 supported carboxylate salts while the culture tubes illustrate the extent of substrate vaporization as a function of temperature (right).

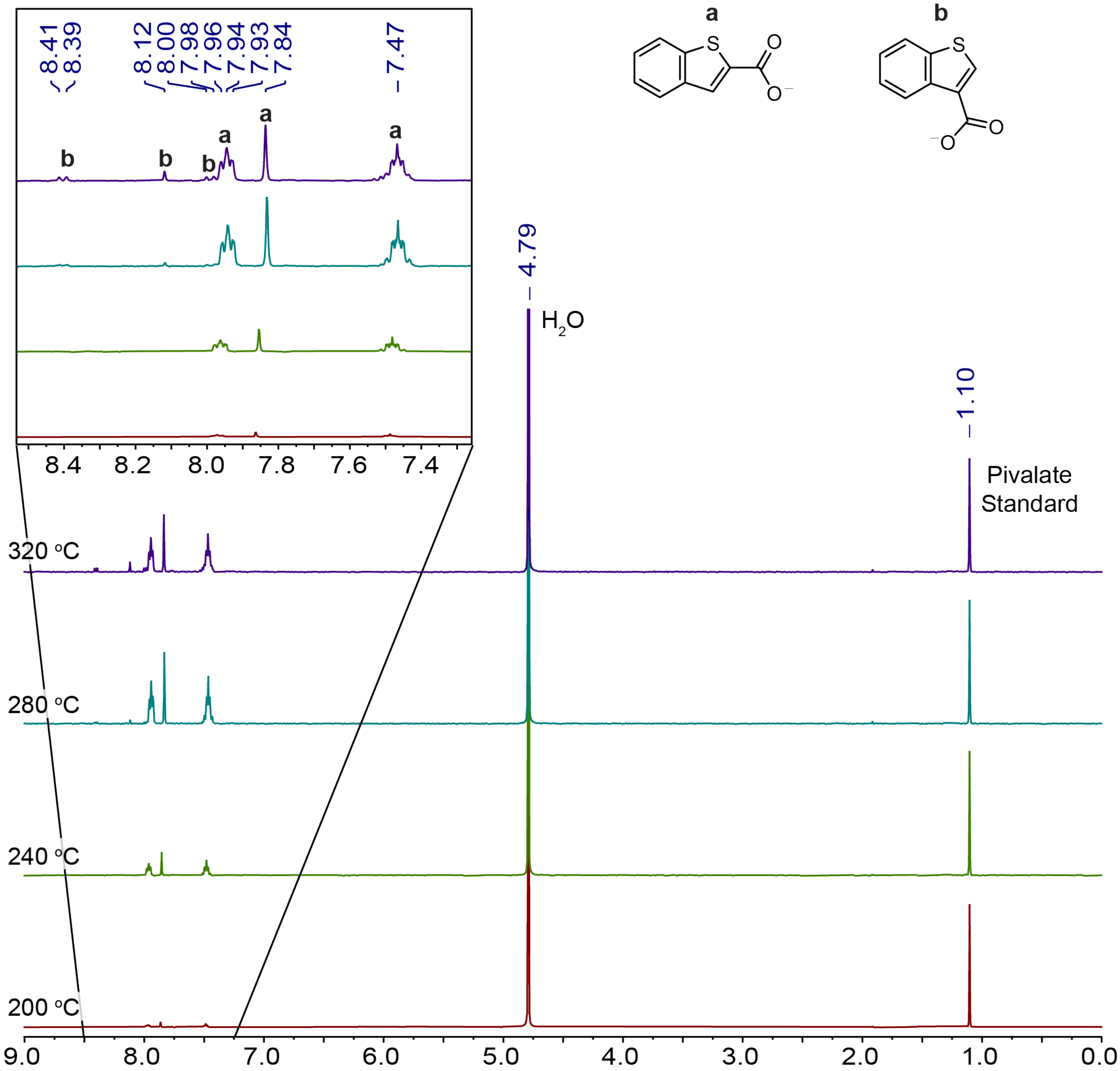

Figure S4. ^{1}H NMR (400 MHz) in D_2O of the extracted product mixture for the benzothiophene carboxylation temperature series with Cs_2CO_3/TiO_2. Sodium pivalate was used as an internal standard.

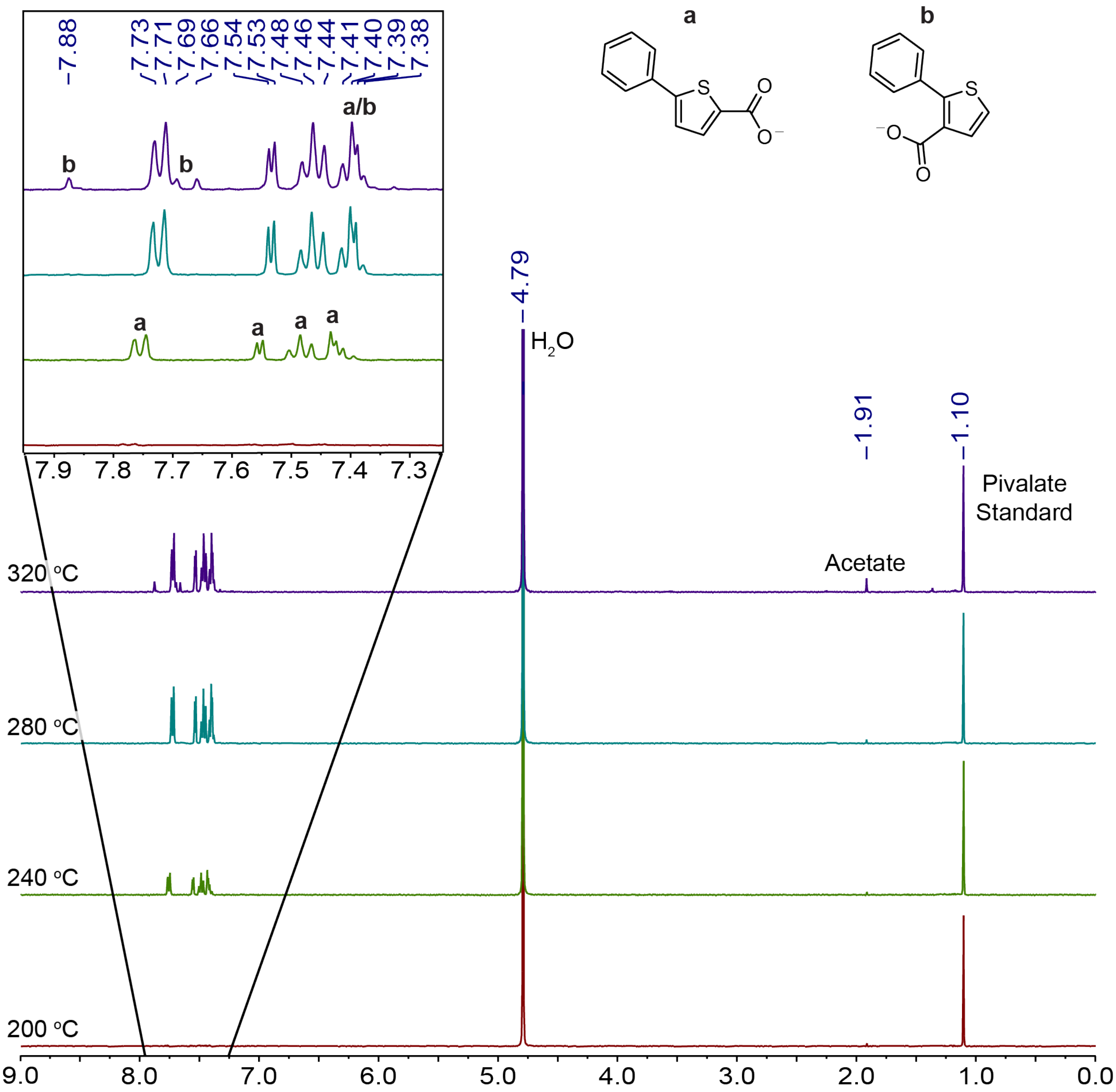

Figure S5. ^{1}H NMR (400 MHz) in D_2O of the extracted product mixture for the phenylthiophene carboxylation temperature series with Cs_2CO_3/TiO_2. Sodium pivalate was used as an internal standard.

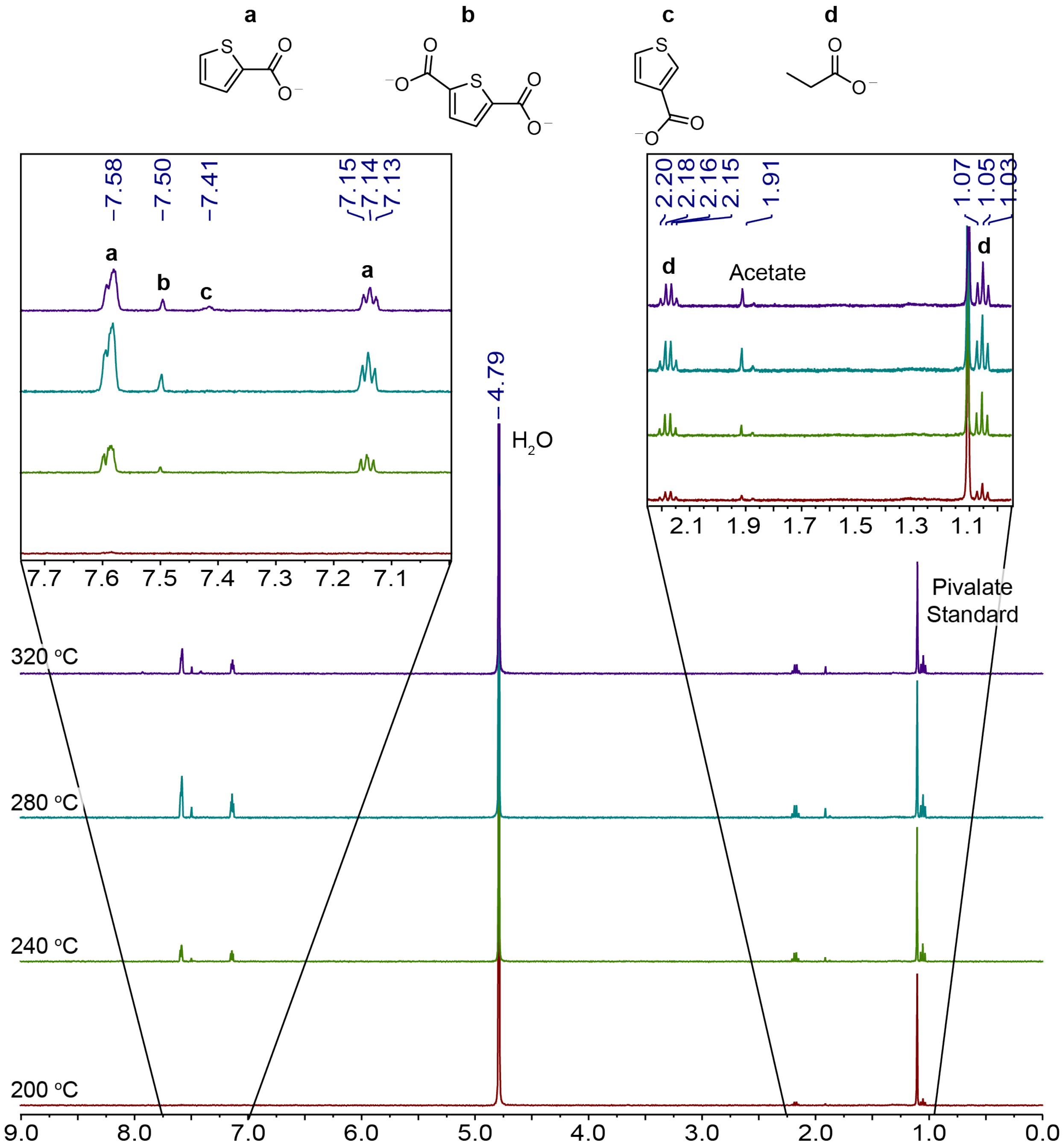

Figure S6. ^{1}H NMR (400 MHz) in D_2O of the extracted product mixture for the thiophene carboxylation temperature series with Cs_2CO_3/TiO_2. Sodium pivalate was used as an internal standard.

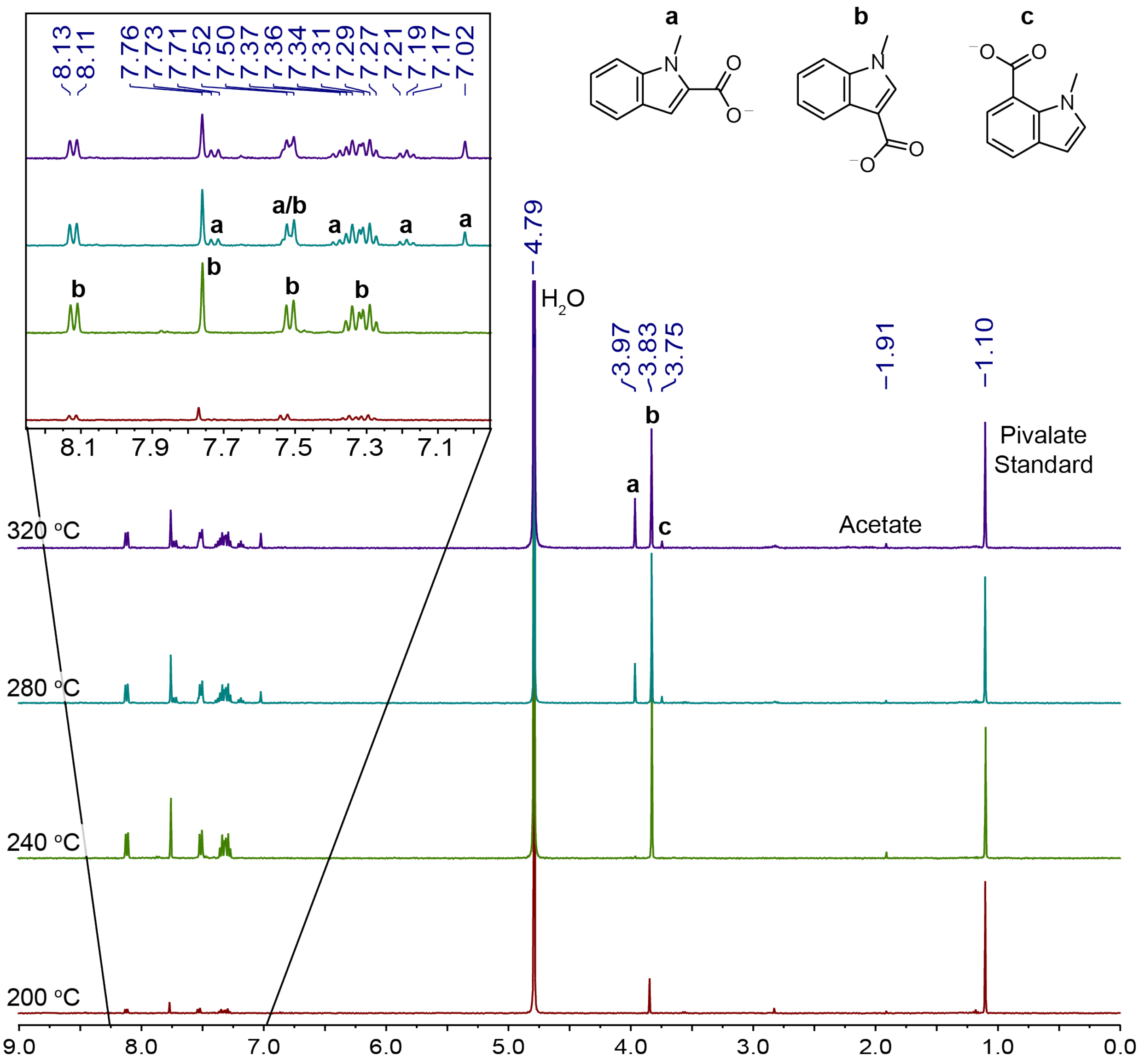

Figure S7. ^{1}H NMR (400 MHz) in D_2O of the extracted product mixture for the 1-methylindole carboxylation temperature series with Cs_2CO_3/TiO_2. Sodium pivalate was used as an internal standard.

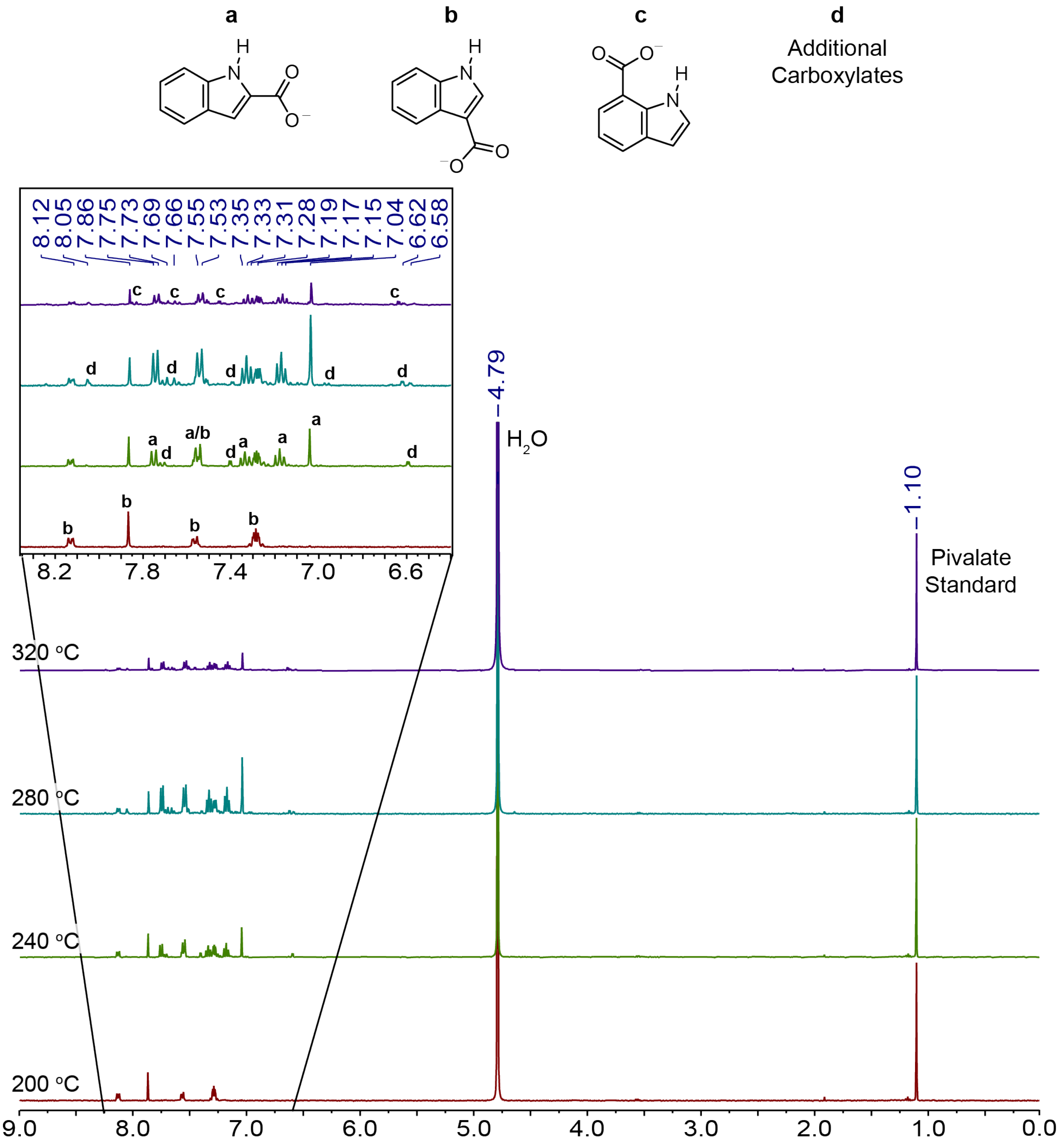

Figure S8. ^{1}H NMR (400 MHz) in D_2O of the extracted product mixture for the indole carboxylation temperature series with Cs_2CO_3/TiO_2. Sodium pivalate was used as an internal standard.

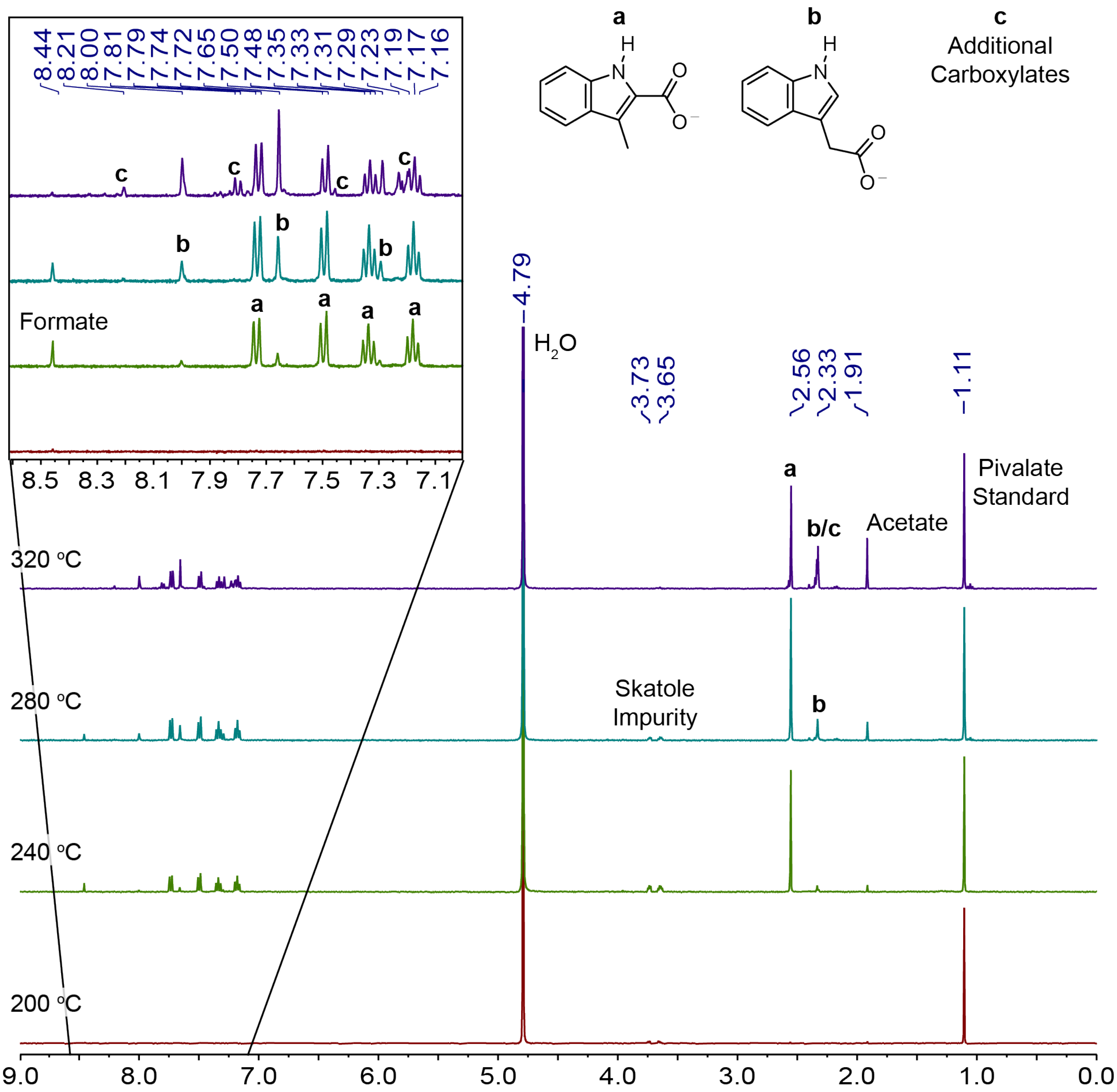

Figure S9. ^{1}H NMR (400 MHz) in D_2O of the extracted product mixture for the skatole carboxylation temperature series with Cs_2CO_3/TiO_2. Sodium pivalate was used as an internal standard.

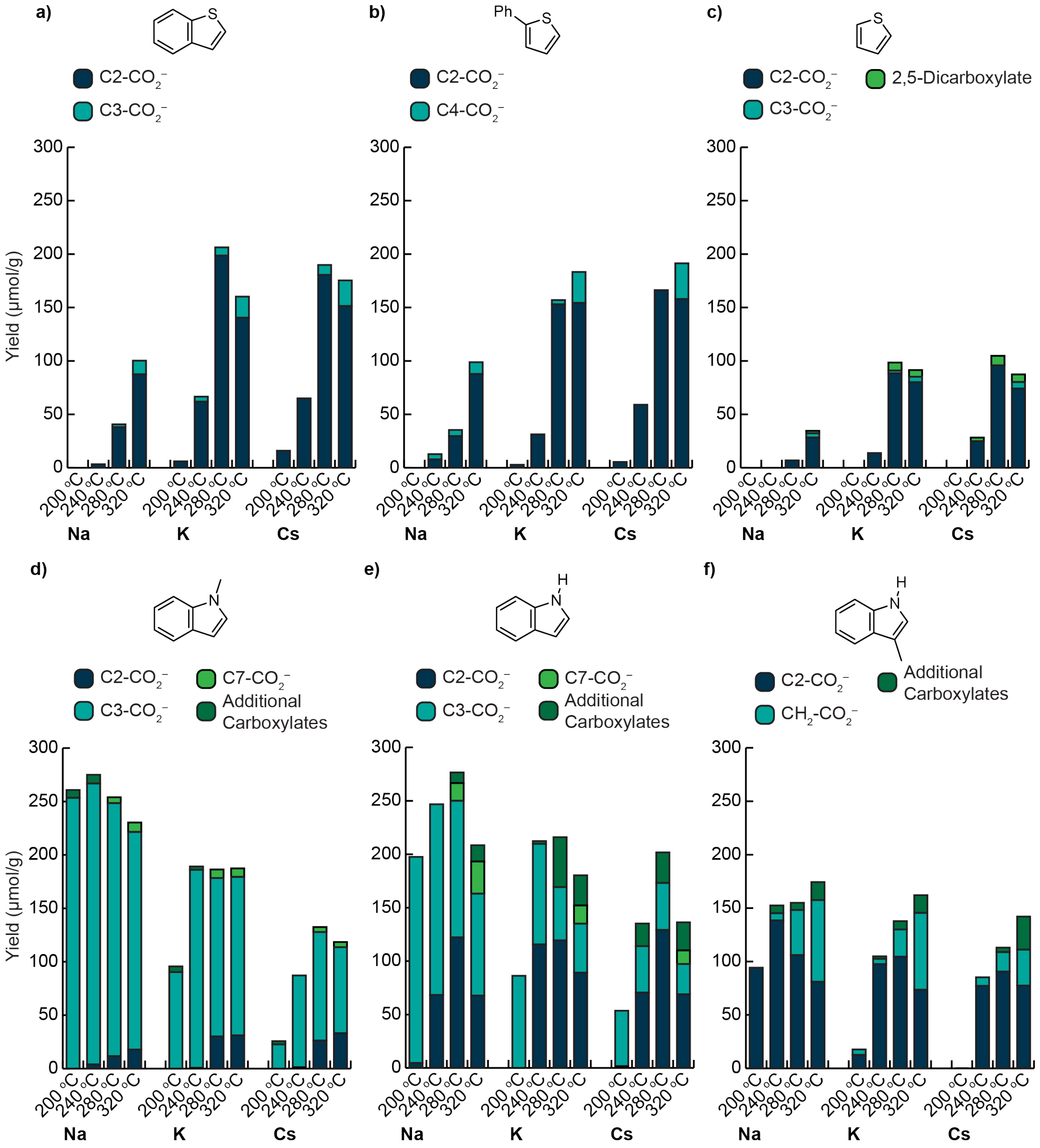

Figure S10. Summary of C–H carboxylation for heteroarenes using M_2CO_3/TiO_2 (M = Na^+, K^+, or Cs^+) and CO_2 at temperatures from 200 °C to 320 °C. The heterocycle substrates were contained in glass culture tubes to separate any un-vaporized substrate from the M_2CO_3/TiO_2 material. Reaction conditions: 250 mg M_2CO_3/TiO_2, 1.5 mmol heterocycle, 2.5 bar (298 K) CO_2, 3 h reaction time.

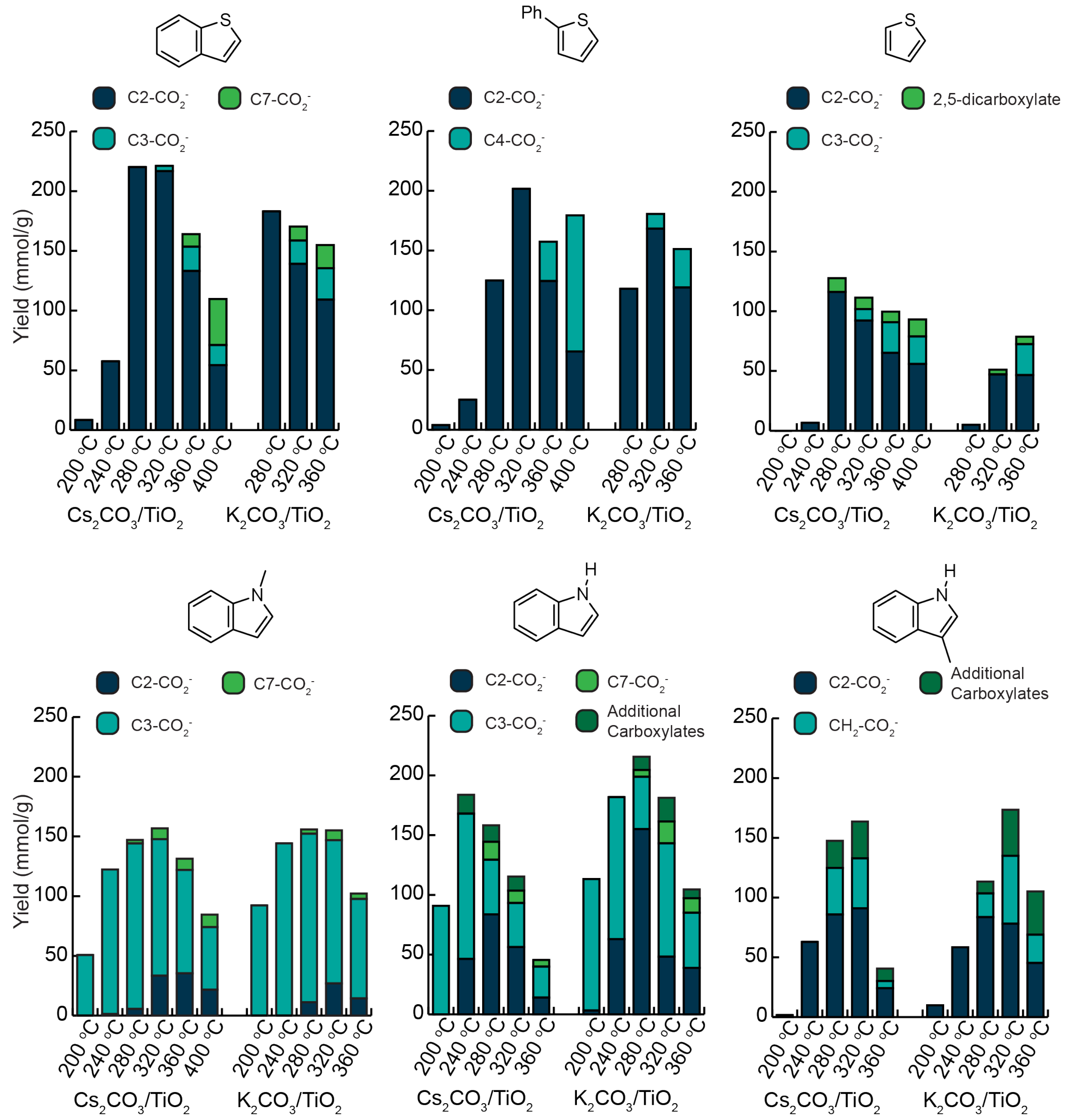

Figure S11. Summary of C–H carboxylation for heteroarenes using M_2CO_3/TiO_2 and CO_2 at varying temperatures without using culture tubes to separate the substrate from the M_2CO_3/TiO_2 material. Reaction conditions: ~250 mg M_2CO_3/TiO_2, ~2.9 mmol heterocycle, and 4.5 bar CO_2 at room temperature.

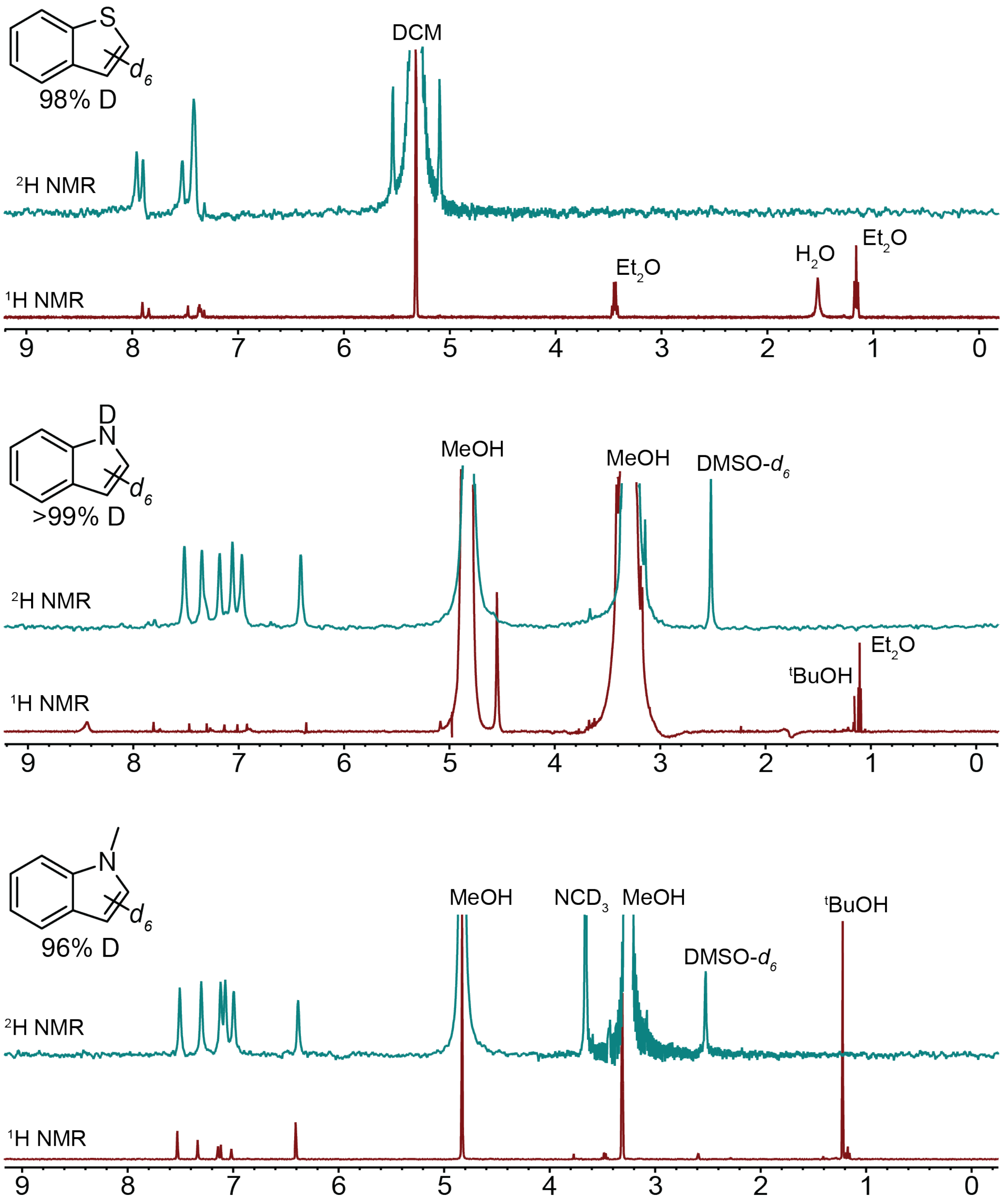

Figure S12. ^{1}H and ^{2}H NMR of benzothiophene-*d*$_6$ (top, DCM-*d*$_2$), indole-*d*$_7$ (middle, MeOD), and methylindole-*d*$_9$ (bottom, MeOD). %Deuteration was determined through integration using an internal standard of ^{t}BuOH for ^{1}H NMR and DMSO-*d*$_6$ for ^{2}H NMR.

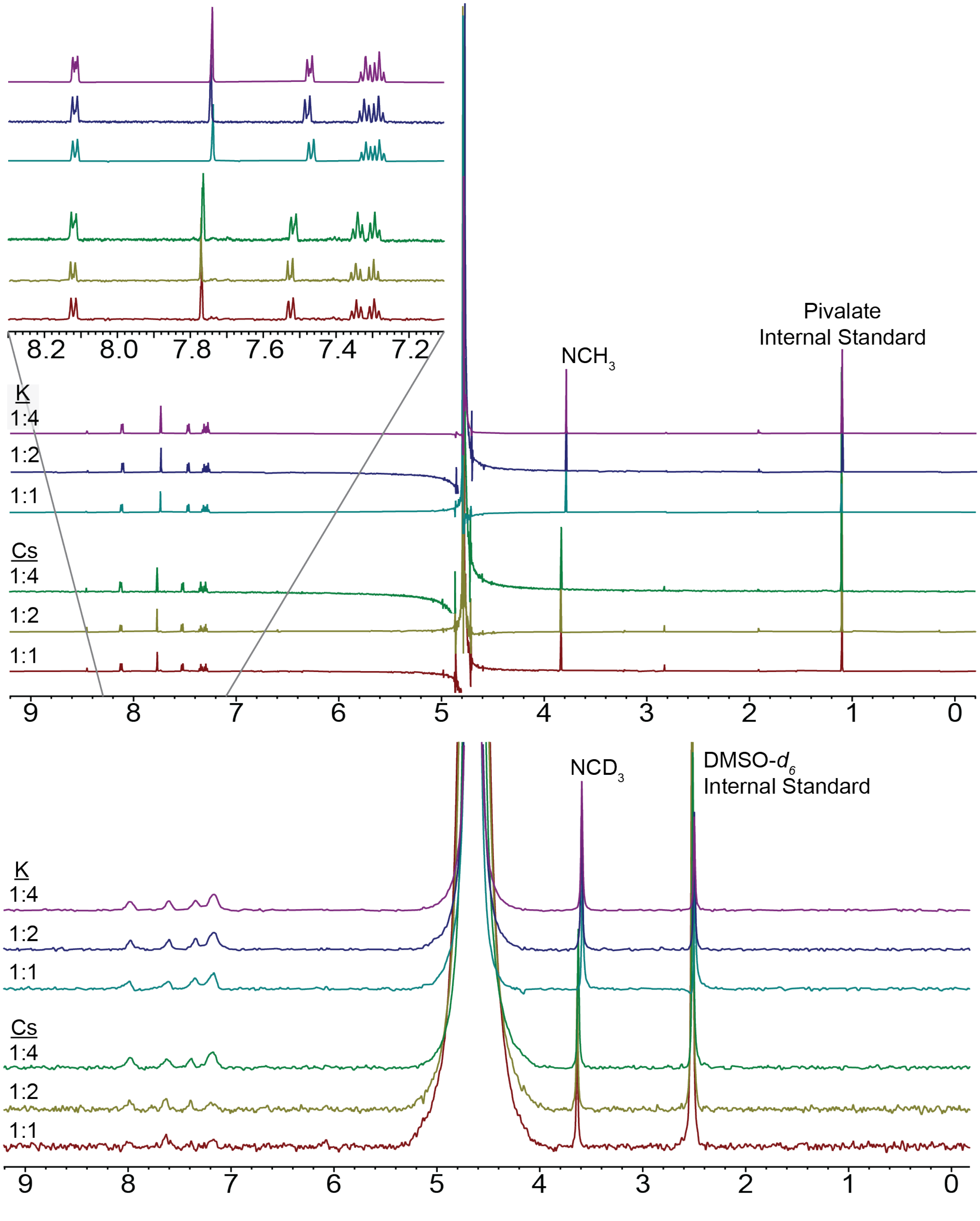

Figure S13. ^{1}H NMR (600 MHz, top) and ^{2}H NMR (600 MHz, bottom) for the kinetic isotope competition experiments with methylindole-*d*$_9$. Experiments were conducted in 9:1 H_2O:D_2O solvent mixtures with water suppression. Sodium pivalate was used as the internal standard for ^{1}H NMR while DMSO-*d*$_6$ was used for ^{2}H NMR.

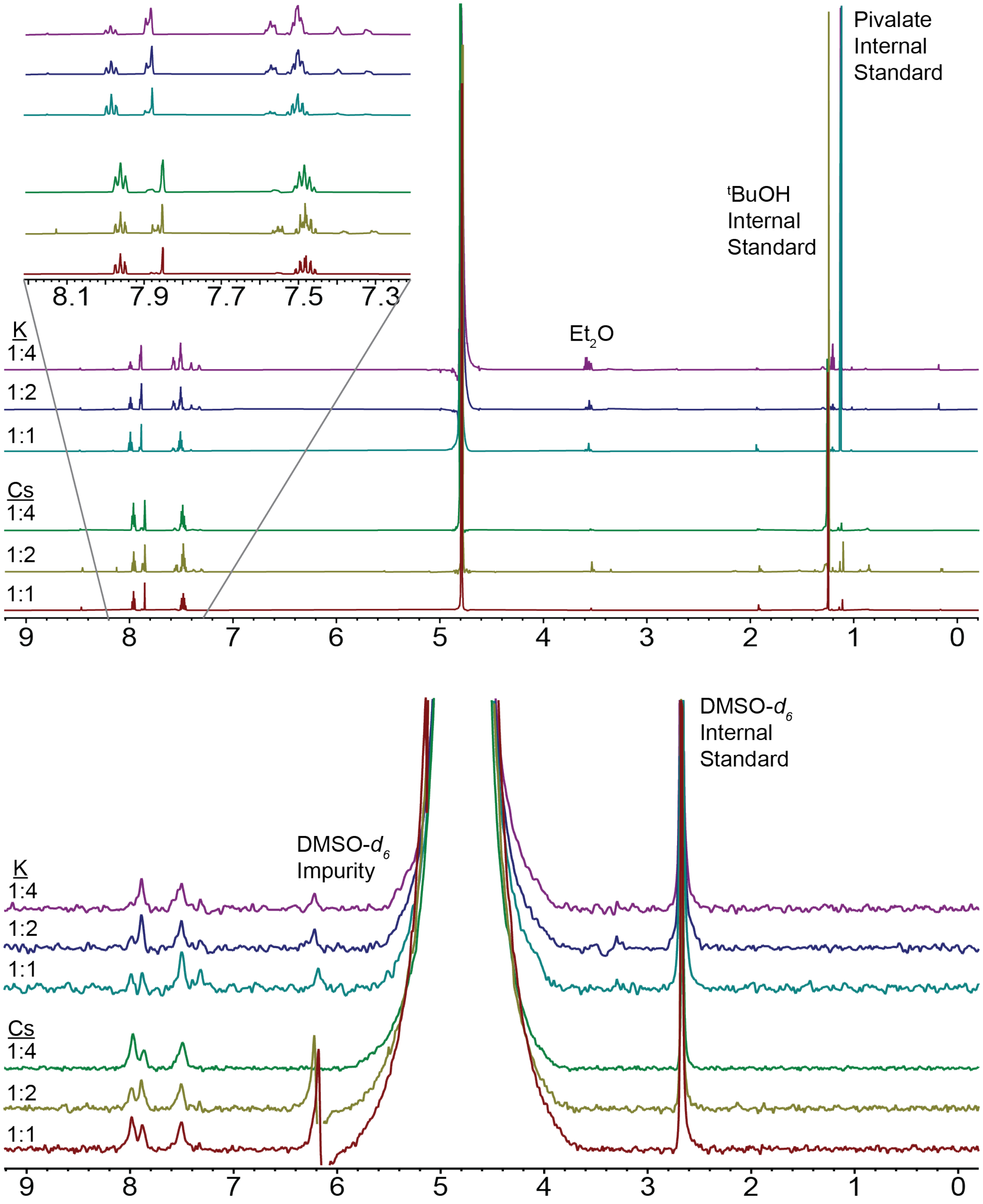

Figure S14. ^{1}H NMR (600 MHz, top) and ^{2}H NMR (600 MHz, bottom) for the kinetic isotope competition experiments for benzothiophene-d_6. Experiments were conducted in 9:1 H_2O:D_2O solvent mixtures with water suppression. ^{t}BuOH was used as the internal standard for Cs_2CO_3 and sodium pivalate was used for K_2CO_3 ^{1}H NMR experiments while DMSO-d_6 was used for all ^{2}H NMR experiments. The extra peaks in the aromatic region of the ^{1}H NMR experiments likely arise from carboxylation of partially deuterated starting material.

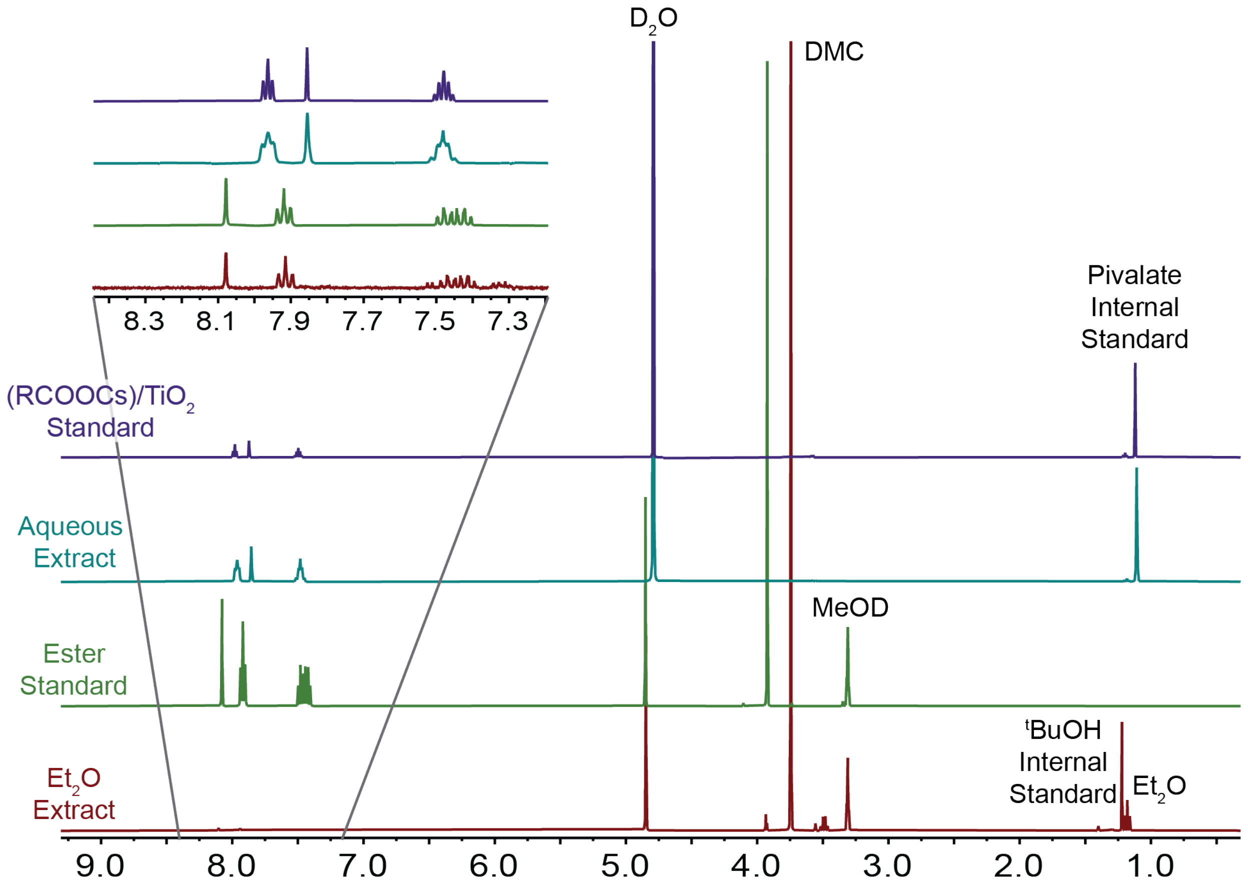

Figure S15. ^{1}H NMR (400 MHz) of the ether extracted product mixture (bottom, red) and the aqueous extract of the TiO_2 support (upper-middle, blue) after a methylation reaction under 8 bar of dimethyl carbonate 120 °C for 6 h. Shown for product validation is a standard NMR of pure methyl benzothiophene-2-carboxylate (lower-middle, green) and an aliquot of the supported carboxylate before methylation (top, purple). For the aqueous extracts pivalate was used as the internal standard while *t*-butanol was used for the ether extracts. Note only a trace amount of ester formation is observed with this procedure.

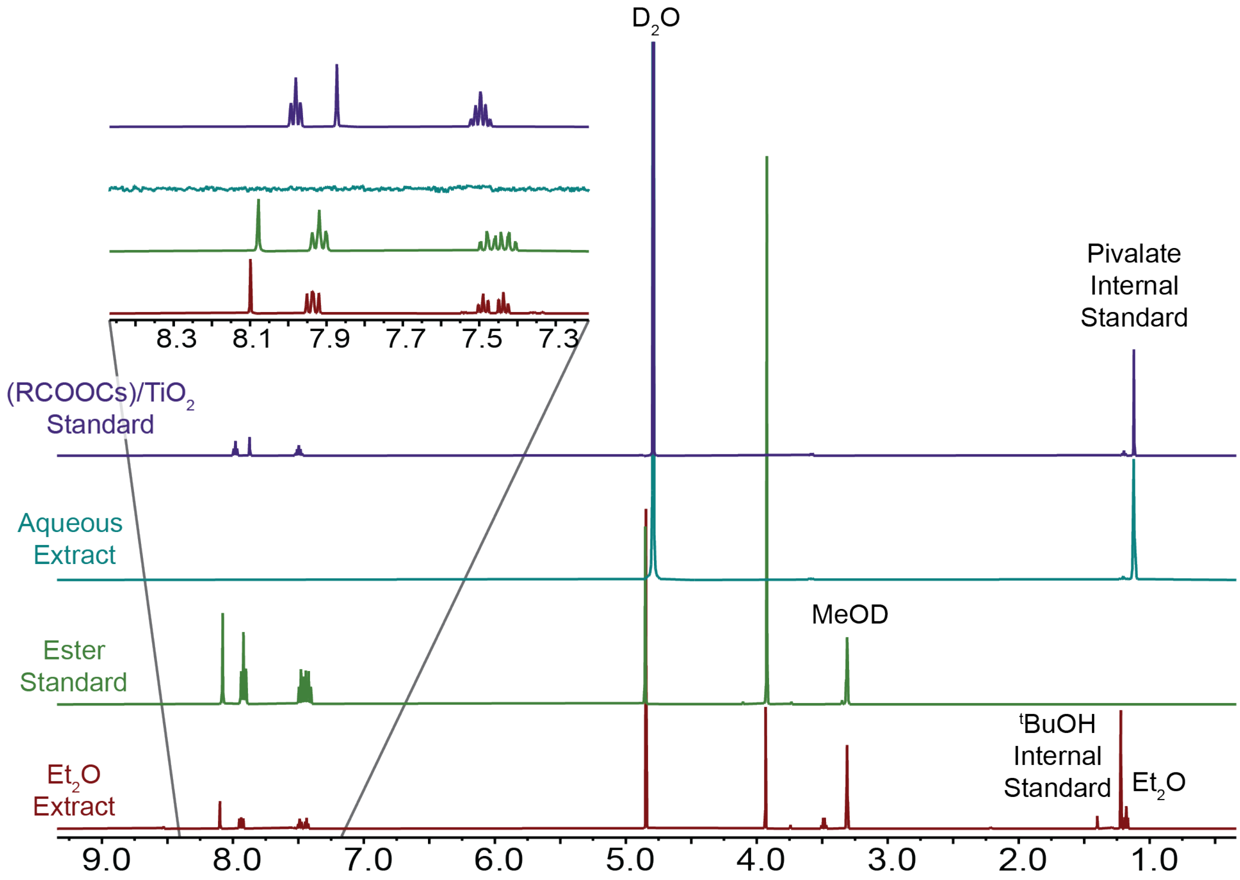

Figure S16. ^{1}H NMR (400 MHz) the ether extracted product mixture (bottom, red) and the aqueous extract of the TiO_2 support (upper-middle, blue) after a methylation reaction under 8 bar of dimethyl carbonate 160 °C for 6 h. Shown for product validation is a standard NMR of pure methyl benzothiophene-2-carboxylate (lower-middle, green) and an aliquot of the supported carboxylate before methylation (top, purple). For the aqueous extracts pivalate was used as the internal standard while *t*-butanol was used for the ether extracts. Note that ester formation is nearly quantitative with this procedure.

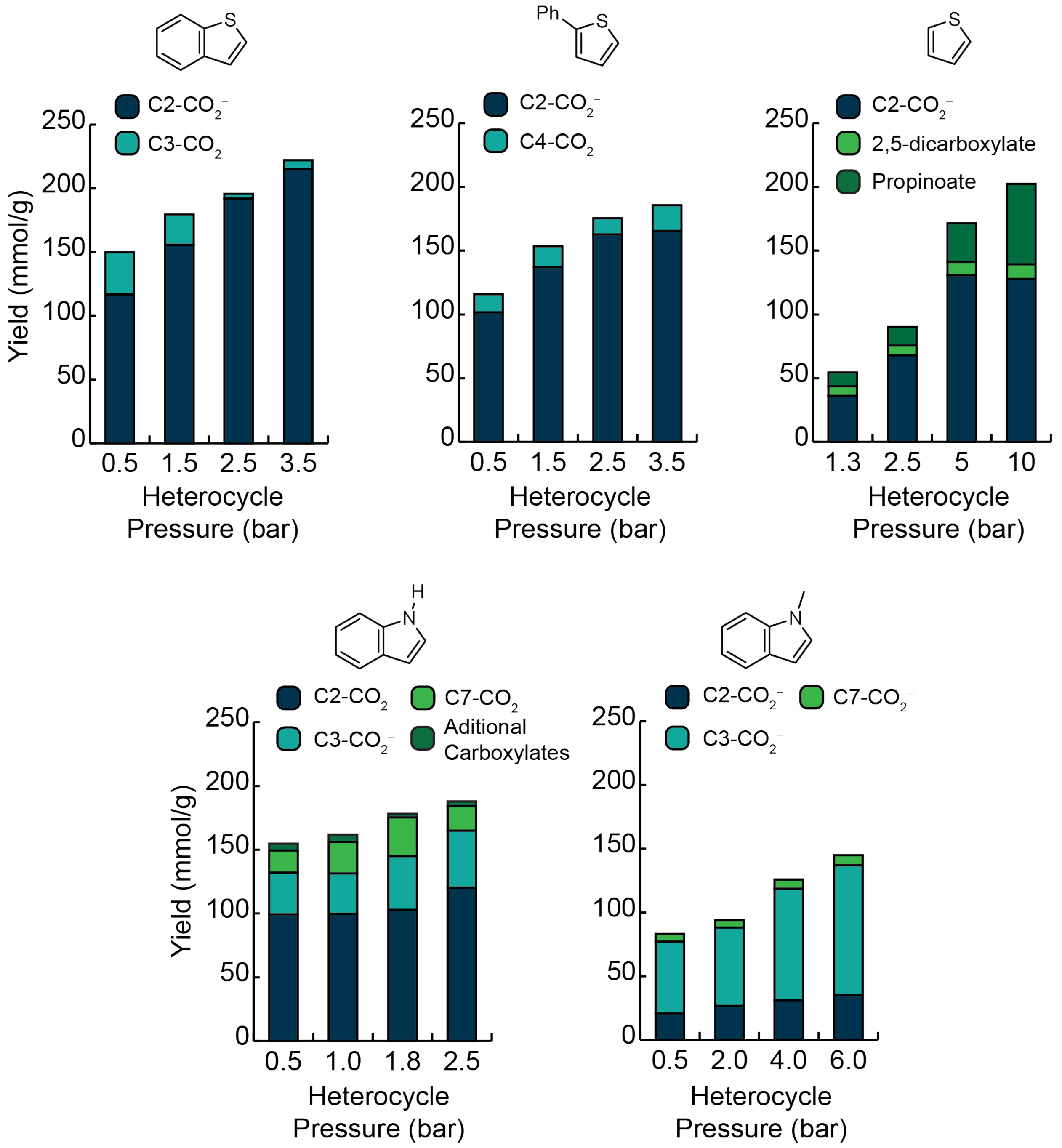

Figure S17. Summary of heteroarene carboxylation yields for Cs_2CO_3/TiO_2 as a function of substrate pressure. Reaction conditions: 250 mg of Cs_2CO_3/TiO_2, 2.0 bar CO_2 at 298 K, and 180 min reaction time. T = 280 °C (benzothiophene, indole, and thiophene) or 320 °C (phenylthiophene and methylindole). The highest pressure data point in each series corresponds to the predicted saturation pressure of the heterocycle (See **Table S1**).

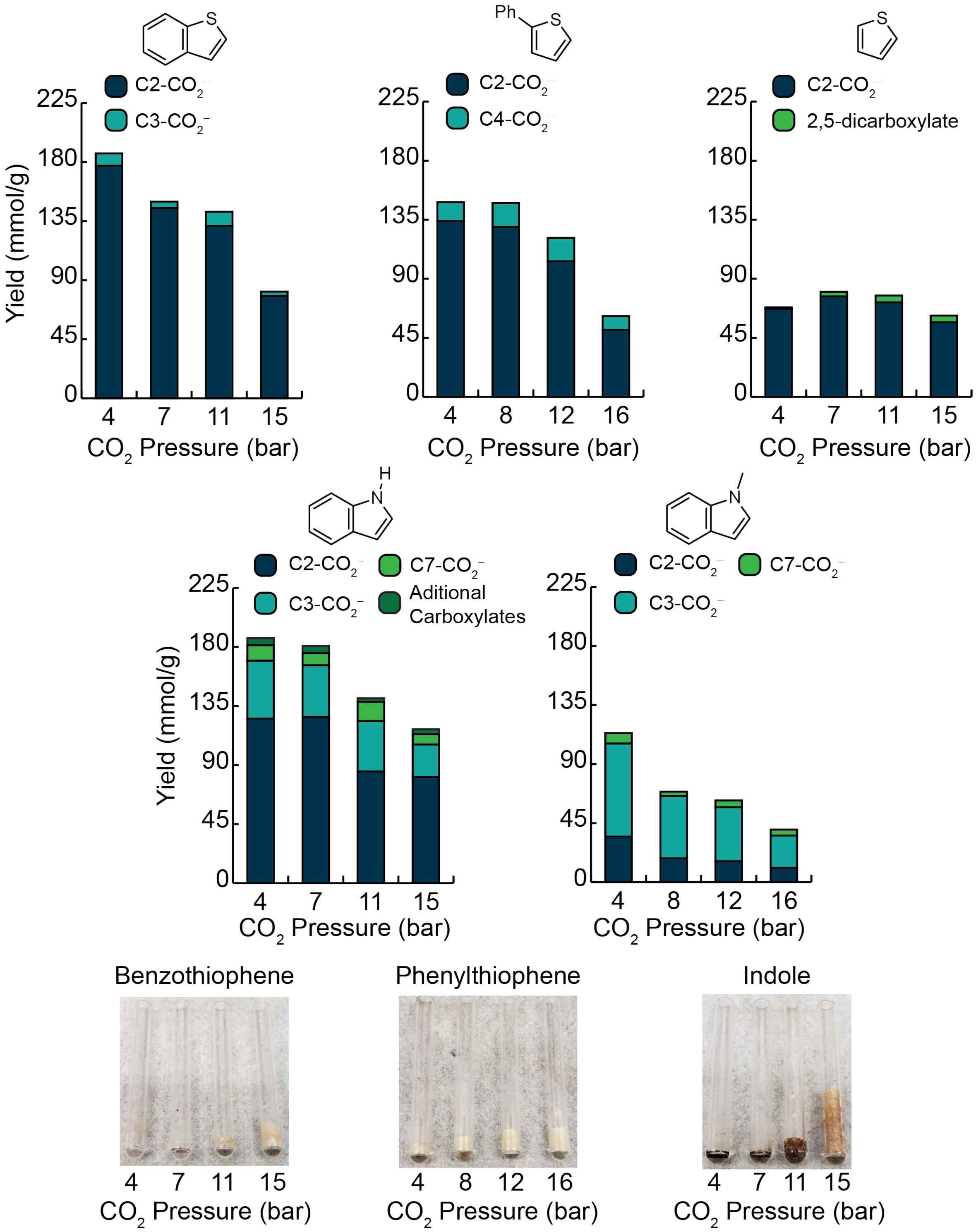

Figure S18. (top) Summary of heteroarene carboxylation yields for Cs_2CO_3/TiO_2 as a function of CO_2 pressure at reaction temperature. Reaction conditions: 200 mg of Cs_2CO_3/TiO_2, 0.97 mmol of heterocycle (1.5 bar at reaction temperature), and 180 min. reaction time. T = 280 °C (benzothiophene, indole, and thiophene) or 320 °C (phenylthiophene and methylindole). (bottom) Un-vaporized heteroarene remaining in culture tubes post carboxylation as a function of CO_2 pressure.

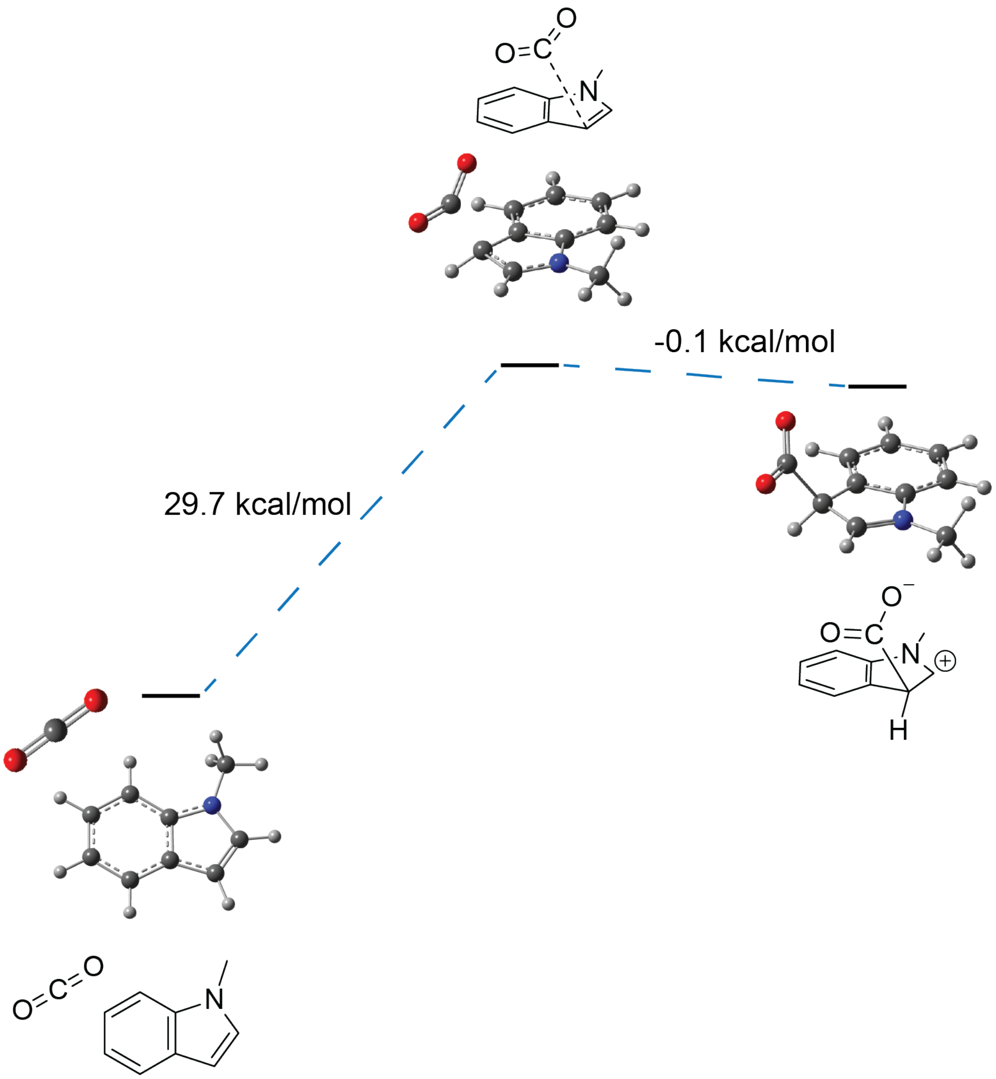

Figure S19. DFT computed free energy diagram for the electrophilic aromatic substitution reaction of 1-methylindole with CO_2 in acetonitrile solvation. All structures have been characterized by frequency calculations as either a minimum (no imaginary modes) or a transition state (one imaginary vibration mode). IRC calculations additionally confirm that the transition state connects both reactant and intermediate structures shown.

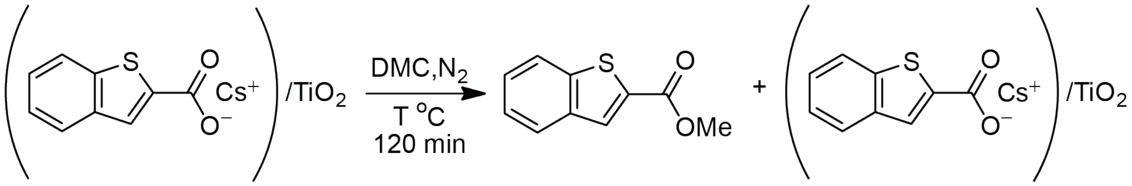

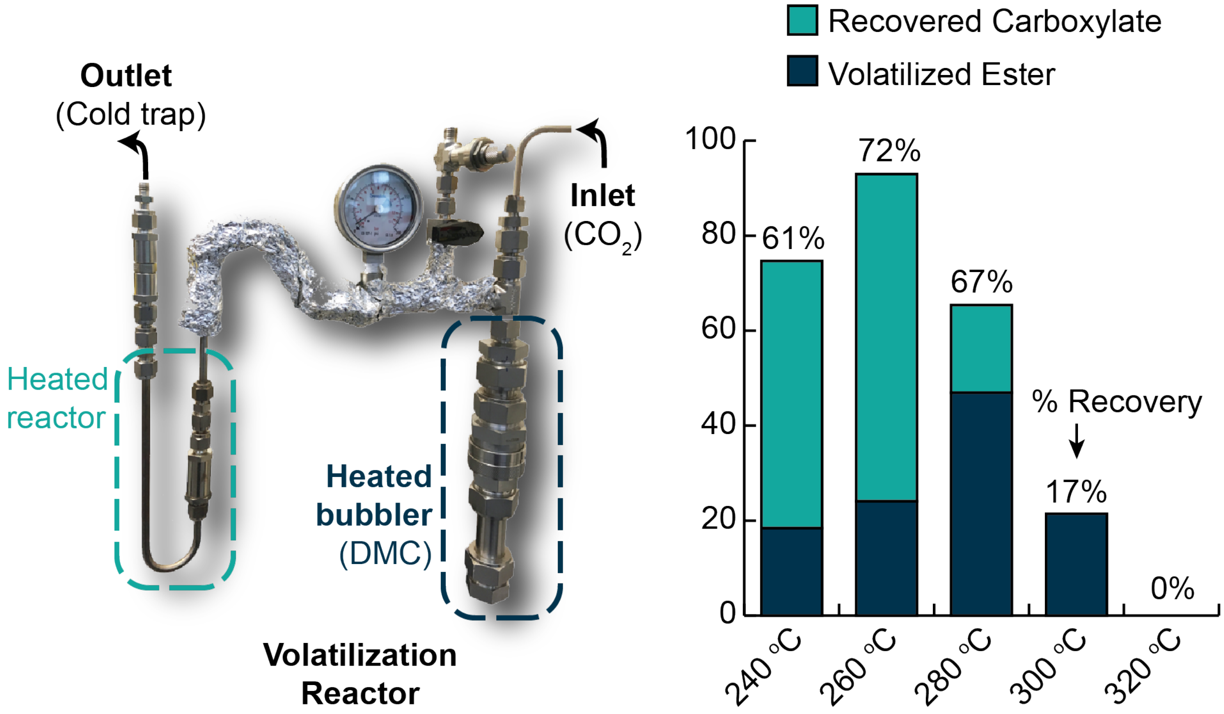

Figure S20. (left) Stainless steel flow reactor setup used for volatilization reactions. A stainless steel bubbler containing dimethyl carbonate (DMC) is heated using insulated heat tape (80–90 °C) to achieve the desired vapor partial pressure. A second strand of heat tape is wrapped around the reactor containing the supported carboxylate material which is then set to the desired temperature (240–320 °C). The total pressure in the reactor is regulated by a downstream check valve and monitored using the pressure gauge. (right) Summary of volatilization results for Cs as a function of temperature. Reaction conditions: ~100 mg of supported carboxylate, 0.3 bar of dimethyl carbonate flowing at 40 mL/min for 120 min. The organics were isolated downstream using a cold trap while un-reacted supported carboxylates were isolated by aqueous extraction of the post-reaction TiO_2 support. Yields were determined by ^{1}H NMR using an internal standard.

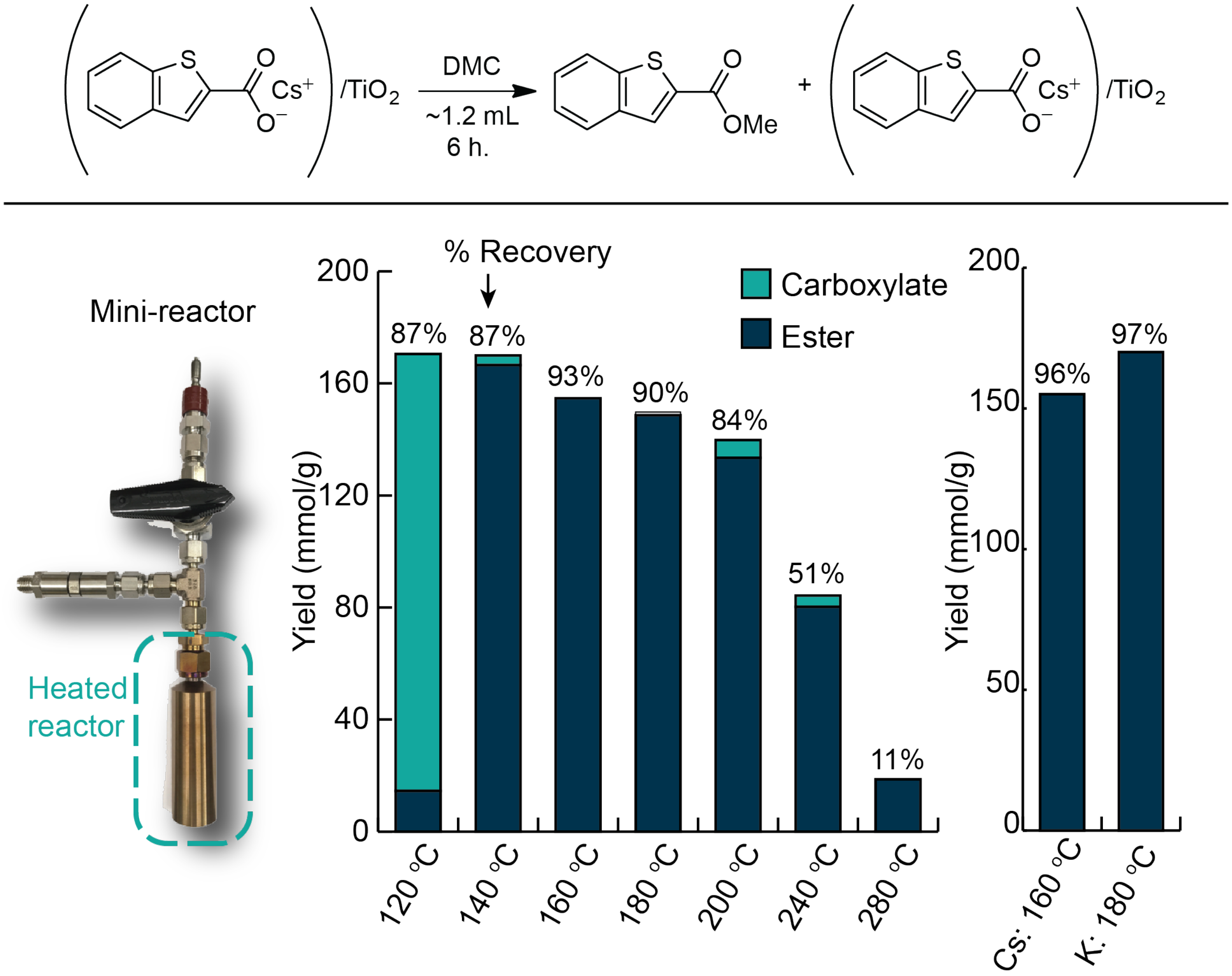

Figure S21. (left) Mini-reactor used for carboxylation/esterification experiments. (Middle) Esterification of dispersed benzothiophene-2-carboxylate as a function of temperature for Cs. Reaction conditions: ~100 mg of dispersed carboxylate ($RCOOCs/TiO_2$) and 1 mL of dimethyl carbonate heated to desired temperature for 6 h under N_2 atmosphere. (right) Scaled esterification of dispersed benzothiophene-2-carboxylate at optimal temperatures for Cs and K. Reaction conditions: ~1.25 g of dispersed carboxylate and 1 mL of dimethyl carbonate heated to specified temperature for 3 h. %Recovery is the sum of both unreacted, supported carboxylate and isolated ester. Ester was isolated by ethyl ether extraction while unreacted, supported carboxylate was isolated by aqueous extraction of the post-reaction TiO_2 support. Both carboxylate and ester were then quantified by ^{1}H NMR.

Table S1. Saturation vapor pressures (bar) of the studied heterocycles as a function of temperature derived from the Clausius-Clapeyron equation.

Substrate	Saturation Vapor Pressure (bar)					
	**200 °C**	**240 °C**	**280 °C**	**320 °C**	**360 °C**	**400 °C**
Benzothiophene	0.61	1.59	3.59	7.25	13.42	23.08
Phenylthiophene	0.28	0.73	1.66	3.35	6.19	10.65
Thiophene	17.11	33.58	59.77	98.44	152.2	223.45
Indole	0.15	0.67	2.38	7.16	18.72	43.66
Methylindole	0.33	1.12	3.21	7.98	17.67	35.59
Skatole	0.14	0.51	1.52	3.93	8.98	18.6

Table S2. Summary of the DFT thermochemical analysis for the electrophilic aromatic substitution reaction of 1-methylindole with CO_2 in solvents with varying dielectric constant.

Solvent	ΔG°_{rxn}(TS) (kcal mol^{-1})	ΔG°_{rxn}(Int) (kcal mol^{-1})	$\Delta\Delta G^{\circ}$(Int–TS) (kcal mol^{-1})
Gas Phase	---	---	---
Benzene (ε = 2.3)	---	---	---
Quinoline (ε = 9.2)	---	---	---
1-Propanol (ε = 20.5)	30.22	29.96	-0.26
Acetonitrile (ε = 32.6)	29.73	29.63	-0.095
DMSO (ε = 46.8)	29.60	29.52	-0.084
Water (ε = 78.4)	29.43	29.35	-0.077

Table S3. Summary of benzothiophene carboxylation yields for M_2CO_3/TiO_2 (M = Na^+, K^+, or Cs^+) as a function of temperature. Yields were determined by aqueous extraction of the carboxylates from the support followed by ^1H NMR analysis. Na_2CO_3/TiO_2 is loaded with 0.56 mmol Na_2CO_3/g TiO_2; K_2CO_3/TiO_2 is loaded with 0.52 mmol K_2CO_3/g TiO_2; and Cs_2CO_3/TiO_2 is loaded with 0.47 mmol Cs_2CO_3/g TiO_2. 100% CO_3^{2-} conversion corresponds to two equivalents of carboxylate formation. Conditions: 250 mg Cs_2CO_3/TiO_2, 1.5 mmol benzothiophene, 2.5 bar CO_2 at 298.15 K, 3 h reaction time.

M_2CO_3 /TiO_2	T (°C)	C2–CO_2^- (µmol/g TiO_2)	C3–CO_2^- (µmol/g TiO_2)	% CO_3^{2-} Conversion
Na	200	0.0	0.0	0.0
	240	3.3	0.0	0.3
	280	38.0	2.7	3.8
	320	87.4	12.9	9.5
K	200	6.0	0.0	0.6
	240	61.8	4.8	6.4
	280	198.6	7.8	19.8
	320	140.4	19.8	15.4
Cs	200	16.0	0.0	1.7
	240	65.0	0.0	6.9
	280	180.5	9.4	20.0
	320	151.3	24.1	18.5

Table S4. Summary of benzothiophene carboxylation yields for M_2CO_3/TiO_2 (M = K^+ or Cs^+) as a function of temperature by direct mixing of the heterocycle and dispersed carbonate. Conditions: 250 mg M_2CO_3/TiO_2, 1.5 mmol benzothiophene, 4.0 bar CO_2 at 298.15 K, and 3 h reaction time.

M_2CO_3 /TiO_2	T (°C)	C2–CO_2^- (µmol/g TiO_2)	C3–CO_2^- (µmol/g TiO_2)	C7–CO_2^- (µmol/g TiO_2)	% CO_3^{2-} Conversion
K	280	183.1	0.0	0.0	17.6
	320	139.1	19.5	11.8	16.4
	360	109.2	26.3	19.5	14.9
Cs	200	8.4	0.0	0.0	0.9
	240	57.6	0.0	0.0	6.1
	280	220.1	0.0	0.0	23.2
	320	216.7	4.5	0.0	23.4
	360	133.2	20.3	10.5	17.3
	400	54.4	16.8	38.5	11.6

Table S5. Summary of benzothiophene carboxylation yields for Cs_2CO_3/TiO_2 as a function of benzothiophene pressure. Yields were determined by aqueous extraction of the carboxylates from the support followed by ^{1}H NMR analysis. Cs_2CO_3/TiO_2 is loaded with 0.47 mmol Cs_2CO_3/g TiO_2; 100% CO_3^{2-} conversion corresponds to two equivalents of carboxylate formation. Conditions: 250 mg Cs_2CO_3/TiO_2, 280 °C, 2.0 bar CO_2 at 298.15 K, and 3 h reaction time.

$P_{benzothiophene}$ (bar)	C2–CO_2^- (μmol/g TiO_2)	C3–CO_2^- (μmol/g TiO_2)	% CO_3^{2-} Conversion
0.5	116.9	33.0	12.9
1.5	155.7	23.8	17.7
2.5	192.1	3.7	20.6
3.5	215.1	7.0	23.5

Table S6. Summary of benzothiophene carboxylation yields for Cs_2CO_3/TiO_2 as a function of CO_2 pressure. Yields were determined by aqueous extraction of the carboxylates from the support followed by ^{1}H NMR analysis. Cs_2CO_3/TiO_2 is loaded with 0.47 mmol Cs_2CO_3/g TiO_2; 100% CO_3^{2-} conversion corresponds to two equivalents of carboxylate formation. Conditions: 250 mg Cs_2CO_3/TiO_2, 280 °C, 0.97 mmol benzothiophene, and 3 h reaction time.

P_{CO2} (bar)	C2–CO_2^- (μmol/g TiO_2)	C3–CO_2^- (μmol/g TiO_2)	% CO_3^{2-} Conversion
4	177.1	9.4	19.6
7	145.0	4.8	15.7
11	131.2	1.0	13.9
15	78.0	3.2	8.5

Table S7. Summary of benzothiophene carboxylation yields for Cs_2CO_3/TiO_2 as a function of Cs_2CO_3/TiO_2 reaction loading. Yields were determined by aqueous extraction of the carboxylates from the support followed by ^{1}H NMR analysis. Cs_2CO_3/TiO_2 is loaded with 0.47 mmol Cs_2CO_3/g TiO_2; 100% CO_3^{2-} conversion corresponds to two equivalents of carboxylate formation. Conditions: 250 mg Cs_2CO_3/TiO_2, 280 °C, 1.5 mmol benzothiophene, 4.0 bar CO_2 at 298.15 K, and 3 h reaction time.

Cs_2CO_3/TiO_2 Loading (mg)	C2–CO_2^- (μmol/g TiO_2)	C3–CO_2^- (μmol/g TiO_2)	% CO_3^{2-} Conversion
125	239.2	3.0	25.6
250	220.1	0.0	23.2
450	228.9	0.0	24.2

Table S8. Summary of 2-phenylthiophene carboxylation yields for M_2CO_3/TiO_2 (M = Na^+, K^+, or Cs^+) as a function of temperature. Yields were determined by aqueous extraction of the carboxylates from the support followed by ^1H NMR analysis. Na_2CO_3/TiO_2 is loaded with 0.56 mmol Na_2CO_3/g TiO_2; K_2CO_3/TiO_2 is loaded with 0.52 mmol K_2CO_3/g TiO_2; and Cs_2CO_3/TiO_2 is loaded with 0.47 mmol Cs_2CO_3/g TiO_2. 100% CO_3^{2-} conversion corresponds to two equivalents of carboxylate formation. Conditions: 250 mg Cs_2CO_3/TiO_2, 1.5 mmol 2-phenylthiophene, 2.5 bar CO_2 at 298.15 K, 3 h reaction time.

**M_2CO_3 /TiO_2**	**T (°C)**	**C2–CO_2^- (µmol/g TiO_2)**	**C4–CO_2^- (µmol/g TiO_2)**	**% CO_3^{2-} Conversion**
Na	200	0.0	0.0	0.0
	240	7.8	5.0	1.2
	280	29.6	5.9	3.4
	320	87.7	11.2	9.3
K	200	2.8	0.0	0.3
	240	31.3	0.0	3.0
	280	153.0	4.0	15.1
	320	154.4	28.9	17.6
Cs	200	5.5	0.0	0.6
	240	59.0	0.0	6.2
	280	166.3	0.0	17.6
	320	157.9	33.5	20.2

Table S9. Summary of 2-phenylthiophene carboxylation yields for M_2CO_3/TiO_2 (M = K^+ or Cs^+) as a function of temperature by direct mixing of the heterocycle and dispersed carbonate. Conditions: 250 mg M_2CO_3/TiO_2, 1.5 mmol 2-phenylthiophene, 4.0 bar CO_2 at 298.15 K, and 1.5 h reaction time.

**M_2CO_3 /TiO_2**	**T (°C)**	**C2–CO_2^- (µmol/g TiO_2)**	**C4–CO_2^- (µmol/g TiO_2)**	**% CO_3^{2-} Conversion**
K	280	118.0	0.0	11.4
	320	168.3	12.3	17.4
	360	119.0	32.3	14.6
Cs	200	3.9	0.0	0.4
	240	25.2	0.0	2.7
	280	125.0	0.0	13.2
	320	201.6	0.0	21.3
	360	124.4	33.0	16.6
	400	65.5	114.0	19.0

Table S10. Summary of 2-phenylthiophene carboxylation yields for Cs_2CO_3/TiO_2 as a function of 2-phenylthiophene pressure. Yields were determined by aqueous extraction of the carboxylates from the support followed by ^1H NMR analysis. Cs_2CO_3/TiO_2 is loaded with 0.47 mmol Cs_2CO_3/g TiO_2; 100% CO_3^{2-} conversion corresponds to two equivalents of carboxylate formation. Conditions: 200 mg Cs_2CO_3/TiO_2, 320 °C, 2.0 bar CO_2 at 298.15 K, and 3 h reaction time.

$P_{phenylthiophene}$ (bar)	C2–CO_2^- (μmol/g TiO_2)	C4–CO_2^- (μmol/g TiO_2)	% CO_3^{2-} Conversion
0.5	101.6	14.2	12.2
1.5	137.2	16.3	16.2
2.5	162.7	12.9	18.5
3.5	165.4	20.2	19.6

Table S11. Summary of 2-phenylthiophene carboxylation yields for Cs_2CO_3/TiO_2 as a function of CO_2 pressure. Yields were determined by aqueous extraction of the carboxylates from the support followed by ^1H NMR analysis. Cs_2CO_3/TiO_2 is loaded with 0.47 mmol Cs_2CO_3/g TiO_2; 100% CO_3^{2-} conversion corresponds to two equivalents of carboxylate formation. Conditions: 200 mg Cs_2CO_3/TiO_2, 320 °C, 0.97 mmol 2-phenylthiophene, and 3 h reaction time.

P_{CO2} (bar)	C2–CO_2^- (μmol/g TiO_2)	C4–CO_2^- (μmol/g TiO_2)	% CO_3^{2-} Conversion
4	134.0	14.4	15.7
8	129.5	18.1	15.6
12	103.3	17.8	12.8
16	51.1	10.5	6.5

Table S12. Summary of thiophene carboxylation yields for M_2CO_3/TiO_2 (M = Na^+, K^+, or Cs^+) as a function of temperature. Yields were determined by aqueous extraction of the carboxylates from the support followed by ^{1}H NMR analysis. Na_2CO_3/TiO_2 is loaded with 0.56 mmol Na_2CO_3/g TiO_2; K_2CO_3/TiO_2 is loaded with 0.52 mmol K_2CO_3/g TiO_2; and Cs_2CO_3/TiO_2 is loaded with 0.47 mmol Cs_2CO_3/g TiO_2. 100% CO_3^{2-} conversion corresponds to two equivalents of carboxylate formation. Conditions: 250 mg Cs_2CO_3/TiO_2, 1.5 mmol thiophene, 2.5 bar CO_2 at 298.15 K, 3 h reaction time.

M_2CO_3 /TiO_2	T (°C)	C2–CO_2^- (μmol/g TiO_2)	C3–CO_2^- (μmol/g TiO_2)	C2,5–diCO_2^- (μmol/g TiO_2)	Propionate (μmol/g TiO_2)	% CO_3^{2-} Conversion
Na	200	0.00	0.00	0.00	26.6	2.5
	240	0.00	0.00	0.00	28.3	2.7
	280	6.95	0.00	0.00	29.7	3.5
	320	28.30	4.00	2.35	32.4	6.3
K	200	0.0	0.0	0.0	6.3	0.6
	240	13.8	0.0	0.0	22.4	3.5
	280	88.4	2.7	7.6	25.9	12.0
	320	80.1	5.2	6.2	25.0	11.2
Cs	200	0.0	0.0	0.0	8.4	0.9
	240	24.8	0.0	3.5	21.6	5.3
	280	95.9	0.0	8.9	18.4	13.0
	320	74.1	6.2	7.3	26.6	12.0

Table S13. Summary of thiophene carboxylation yields for M_2CO_3/TiO_2 (M = K^+ or Cs^+) as a function of temperature by direct mixing of the heterocycle and dispersed carbonate. Conditions: 250 mg M_2CO_3/TiO_2, 1.5 mmol thiophene, 4.0 bar CO_2 at 298.15 K, and 1.5 h reaction time.

M_2CO_3 /TiO_2	T (°C)	C2–CO_2^- (μmol/g TiO_2)	C3–CO_2^- (μmol/g TiO_2)	C2,5–diCO_2^- (μmol/g TiO_2)	Propionate (μmol/g TiO_2)	% CO_3^{2-} Conversion
K	280	4.9	0.0	0.0	12.9	1.7
	320	47.1	0.0	4.0	15.6	6.4
	360	46.6	25.8	6.3	16.0	9.1
Cs	160	0.0	0.0	0.0	0.0	0.0
	200	0.0	0.0	0.0	0.0	0.0
	240	6.6	0.0	0.0	0.0	0.7
	280	116.2	0.0	11.5	4.1	13.9
	320	92.2	9.6	9.6	14.5	13.3
	360	65.1	25.7	8.9	21.0	12.7
	400	55.8	23.0	14.2	22.1	12.2

Table S14. Summary of thiophene carboxylation yields for Cs_2CO_3/TiO_2 as a function of thiophene pressure. Yields were determined by aqueous extraction of the carboxylates from the support followed by ^{1}H NMR analysis. Cs_2CO_3/TiO_2 is loaded with 0.47 mmol Cs_2CO_3/g TiO_2; 100% CO_3^{2-} conversion corresponds to two equivalents of carboxylate formation. Conditions: 200 mg Cs_2CO_3/TiO_2, 280 °C, 2.0 bar CO_2 at 298.15 K, and 3 h reaction time.

$P_{Thiophene}$ (bar)	C2–CO_2^- (μmol/g TiO_2)	C3–CO_2^- (μmol/g TiO_2)	C2,5-diCO_2^- (μmol/g TiO_2)	Propionate (μmol/g TiO_2)	% CO_3^{2-} Conversion
1.3	36.1	0.0	7.6	10.8	5.8
2.5	67.9	0.0	7.8	14.5	9.5
5.0	130.7	0.0	10.5	30.2	18.1
10.0	127.8	0.0	11.4	63.1	21.4

Table S15. Summary of thiophene carboxylation yields for Cs_2CO_3/TiO_2 as a function of CO_2 pressure. Yields were determined by aqueous extraction of the carboxylates from the support followed by ^{1}H NMR analysis. Cs_2CO_3/TiO_2 is loaded with 0.47 mmol Cs_2CO_3/g TiO_2; 100% CO_3^{2-} conversion corresponds to two equivalents of carboxylate formation. Conditions: 200 mg Cs_2CO_3/TiO_2, 1.5 mmol thiophene, 280 °C, and 3 h reaction time.

P_{CO2} (bar)	C2–CO_2^- (μmol/g TiO_2)	C3–CO_2^- (μmol/g TiO_2)	C2,5–diCO_2^- (μmol/g TiO_2)	Propionate (μmol/g TiO_2)	% CO_3^{2-} Conversion
4.0	67.13	0.00	1.07	38.1	11.2
7.0	76.53	0.00	3.53	32.9	11.9
11.0	72.00	0.00	5.17	32.3	11.6
14.0	56.80	0.00	5.13	29.1	9.6

Table S16. Summary of indole carboxylation yields for M_2CO_3/TiO_2 (M = Na^+, K^+, or Cs^+) as a function of temperature. Yields were determined by aqueous extraction of the carboxylates from the support followed by ^1H NMR analysis. Na_2CO_3/TiO_2 is loaded with 0.56 mmol Na_2CO_3/g TiO_2; K_2CO_3/TiO_2 is loaded with 0.52 mmol K_2CO_3/g TiO_2; and Cs_2CO_3/TiO_2 is loaded with 0.47 mmol Cs_2CO_3/g TiO_2. 100% CO_3^{2-} conversion corresponds to two equivalents of carboxylate formation. Conditions: 250 mg Cs_2CO_3/TiO_2, 1.5 mmol indole, 2.5 bar CO_2 at 298.15 K, 3 h reaction time.

M_2CO_3 /TiO_2	T (°C)	C2–CO_2^- (µmol/g TiO_2)	C3–CO_2^- (µmol/g TiO_2)	C7–CO_2^- (µmol/g TiO_2)	Other-CO_2^- (µmol/g TiO_2)	% CO_3^{2-} Conversion
Na	200	4.7	192.9	0.0	0.0	18.7
	240	68.3	178.6	0.0	0.0	23.3
	280	122.2	127.9	16.6	9.9	26.2
	320	67.8	95.3	30.2	15.2	19.7
K	200	0.0	86.3	0.0	0.0	8.3
	240	115.6	94.1	2.7	0.0	20.4
	280	119.3	50.0	21.2	25.5	20.8
	320	89.1	45.9	17.1	28.2	17.3
Cs	200	1.7	51.9	0.0	0.0	5.7
	240	70.5	43.5	18.2	3.0	14.3
	280	129.1	44.0	15.3	13.4	21.3
	320	69.0	28.3	12.7	26.3	14.4

Table S17. Summary of indole carboxylation yields for M_2CO_3/TiO_2 (M = K^+ or Cs^+) as a function of temperature by direct mixing of the heterocycle and dispersed carbonate. Conditions: 250 mg M_2CO_3/TiO_2, 1.5 mmol indole, 4.0 bar CO_2 at 298.15 K, and 3 h reaction time.

M_2CO_3 /TiO_2	T (°C)	C2–CO_2^- (µmol/g TiO_2)	C3–CO_2^- (µmol/g TiO_2)	C7–CO_2^- (µmol/g TiO_2)	Other-CO_2^- (µmol/g TiO_2)	% CO_3^{2-} Conversion
K	200	3.2	110.1	0.0	0.0	10.9
	240	63.0	118.8	0.0	0.0	17.5
	280	155.1	43.7	5.7	11.1	20.7
	320	48.3	94.9	18.2	19.9	17.4
	360	38.9	46.2	12.1	7.4	10.1
	400	3.2	110.1	0.0	0.0	10.9
Cs	200	0.0	90.9	0.0	0.0	9.6
	240	46.4	121.6	0.0	15.8	19.4
	280	83.7	45.7	15.0	13.9	16.7
	320	56.3	36.9	10.4	11.8	12.2
	360	14.1	25.9	5.6	0.0	4.8

Table S18. Summary of indole carboxylation yields for Cs_2CO_3/TiO_2 as a function of indole pressure. Yields were determined by aqueous extraction of the carboxylates from the support followed by ^{1}H NMR analysis. Cs_2CO_3/TiO_2 is loaded with 0.47 mmol Cs_2CO_3/g TiO_2; 100% CO_3^{2-} conversion corresponds to two equivalents of carboxylate formation. Conditions: 250 mg Cs_2CO_3/TiO_2, 280 °C, 2.0 bar CO_2 at 298.15 K, and 3 h reaction time.

P_{Indole} (bar)	C2–CO_2^- (μmol/g TiO_2)	C3–CO_2^- (μmol/g TiO_2)	C7–CO_2^- (μmol/g TiO_2)	Other–CO_2^- (μmol/g TiO_2)	% CO_3^{2-} Conversion
0.5	99.3	32.8	17.3	0.0	15.8
1.1	99.6	31.8	24.7	0.0	16.5
1.8	102.9	42.0	30.5	0.0	18.5
2.5	120.3	44.6	19.2	0.0	19.4

Table S19. Summary of indole carboxylation yields for Cs_2CO_3/TiO_2 as a function of CO_2 pressure. Yields were determined by aqueous extraction of the carboxylates from the support followed by ^{1}H NMR analysis. Cs_2CO_3/TiO_2 is loaded with 0.47 mmol Cs_2CO_3/g TiO_2; 100% CO_3^{2-} conversion corresponds to two equivalents of carboxylate formation. Conditions: 250 mg Cs_2 CO_3/TiO_2, 280 °C, 1.5 mmol indole, and 3 h reaction time.

P_{CO2} (bar)	C2–CO_2^- (μmol/g TiO_2)	C3–CO_2^- (μmol/g TiO_2)	C7–CO_2^- (μmol/g TiO_2)	Other–CO_2^- (μmol/g TiO_2)	% CO_3^{2-} Conversion
4	125.3	44.3	11.7	5.4	19.7
7	126.6	39.4	9.2	5.7	19.1
11	85.1	38.5	14.5	2.8	14.9
15	80.9	24.7	7.9	3.8	12.4

Table S20. Summary of 1-methylindole carboxylation yields for M_2CO_3/TiO_2 (M = Na^+, K^+, or Cs^+) as a function of temperature. Yields were determined by aqueous extraction of the carboxylates from the support followed by ^1H NMR analysis. Na_2CO_3/TiO_2 is loaded with 0.56 mmol Na_2CO_3/g TiO_2; K_2CO_3/TiO_2 is loaded with 0.52 mmol K_2CO_3/g TiO_2; and Cs_2CO_3/TiO_2 is loaded with 0.47 mmol Cs_2CO_3/g TiO_2. 100% CO_3^{2-} conversion corresponds to two equivalents of carboxylate formation. Conditions: 250 mg Cs_2CO_3/TiO_2, 1.5 mmol 1-methylindole, 2.5 bar CO_2 at 298.15 K, 3 h reaction time.

**M_2CO_3 /TiO_2**	**T (°C)**	**C2–CO_2^- (µmol/g TiO_2)**	**C3–CO_2^- (µmol/g TiO_2)**	**C7–CO_2^- (µmol/g TiO_2)**	**Other-CO_2^- (µmol/g TiO_2)**	**% CO_3^{2-} Conversion**
Na	200	0.0	253.4	0.0	7.4	24.7
	240	3.9	263.0	0.0	8.2	26.0
	280	11.6	236.9	5.5	0.0	24.0
	320	17.8	203.7	8.9	0.0	21.8
K	200	0.0	90.3	0.0	5.4	9.2
	240	0.7	185.4	0.0	3.1	18.2
	280	30.0	148.4	8.0	0.0	17.9
	200	0.0	253.4	0.0	7.4	24.7
Cs	240	3.9	263.0	0.0	8.2	26.0
	280	11.6	236.9	5.5	0.0	24.0
	320	17.8	203.7	8.9	0.0	21.8
	200	0.0	90.3	0.0	5.4	9.2

Table S21. Summary of 1-methylindole carboxylation yields for M_2CO_3/TiO_2 (M = K^+ or Cs^+) as a function of temperature by direct mixing of the heterocycle and dispersed carbonate. Conditions: 250 mg M_2CO_3/TiO_2, 1.5 mmol 1-methylindole, 4.0 bar CO_2 at 298.15 K, and 3 h reaction time.

**M_2CO_3 /TiO_2**	**T (°C)**	**C2–CO_2^- (µmol/g TiO_2)**	**C3–CO_2^- (µmol/g TiO_2)**	**C7–CO_2^- (µmol/g TiO_2)**	**Other-CO_2^- (µmol/g TiO_2)**	**% CO_3^{2-} Conversion**
K	200	0	92.3	0	0.0	8.9
	240	0	144.2	0	0.0	13.9
	280	11.2	141.1	3.6	0.0	15.0
	320	27	119.9	8.2	0.0	14.9
	360	14.4	83.2	4.6	0.0	9.8
	400	0	92.3	0	0.0	8.9
Cs	200	0	50.7	0	0.0	5.4
	240	1.3	121.1	0	0.0	12.9
	280	5.6	138.5	3	0.0	15.5
	320	33.4	114.3	9.1	0.0	16.6
	360	35.4	86.5	9.4	0.0	13.9
	400	21.7	52.4	10.4	0.0	8.9

Table S22. Summary of 1-methylindole carboxylation yields for Cs_2CO_3/TiO_2 as a function of indole pressure. Yields were determined by aqueous extraction of the carboxylates from the support followed by ^1H NMR analysis. Cs_2CO_3/TiO_2 is loaded with 0.47 mmol Cs_2CO_3/g TiO_2; 100% CO_3^{2-} conversion corresponds to two equivalents of carboxylate formation. Conditions: 250 mg Cs_2CO_3/TiO_2, 320 °C, 2.0 bar CO_2 at 298.15 K, and 3 h reaction time.

$P_{Methylindole}$ (bar)	C2–CO_2^- (μmol/g TiO_2)	C3–CO_2^- (μmol/g TiO_2)	C7–CO_2^- (μmol/g TiO_2)	Other–CO_2^- (μmol/g TiO_2)	% CO_3^{2-} Conversion
0.5	20.9	56.4	5.9	0.0	8.8
2.0	26.7	61.4	6.0	0.0	9.9
4.0	31.1	87.6	7.1	0.0	13.3
6.0	35.3	101.7	8.0	0.0	15.3

Table S23. Summary of 1-methylindole carboxylation yields for Cs_2CO_3/TiO_2 as a function of CO_2 pressure. Yields were determined by aqueous extraction of the carboxylates from the support followed by ^1H NMR analysis. Cs_2CO_3/TiO_2 is loaded with 0.47 mmol Cs_2CO_3/g TiO_2; 100% CO_3^{2-} conversion corresponds to two equivalents of carboxylate formation. Conditions: 250 mg Cs_2CO_3/TiO_2, 320 °C, 1.5 mmol 1-methylindole, and 3 h reaction time.

P_{CO2} (bar)	C2–CO_2^- (μmol/g TiO_2)	C3–CO_2^- (μmol/g TiO_2)	C7–CO_2^- (μmol/g TiO_2)	Other–CO_2^- (μmol/g TiO_2)	% CO_3^{2-} Conversion
4	34.7	71.0	7.9	0.0	12.0
8	18.1	47.5	3.3	0.0	7.3
12	15.9	41.2	5.2	0.0	6.6
16	10.8	24.7	4.5	0.0	4.2

Table S24. Summary of skatole carboxylation yields for Cs_2CO_3/TiO_2 as a function of temperature. Yields were determined by aqueous extraction of the carboxylates from the support followed by ^1H NMR analysis. $Cs_2CO_3/TiO_2$is loaded with 0.47 mmol Cs_2CO_3/g TiO_2; 100% CO_3^{2-} conversion corresponds to two equivalents of carboxylate formation. Conditions: 250 mg Cs_2CO_3/TiO_2, 1.5 mmol skatole, 2.5 bar CO_2 at 298.15 K, and 3 h reaction time.

M_2CO_3 /TiO_2	T (°C)	C2–CO_2^- (μmol/g TiO_2)	CH_2–CO_2^- (μmol/g TiO_2)	Other–CO_2^- (μmol/g TiO_2)	% CO_3^{2-} Conversion
Na	200	94.2	0.0	0.0	8.9
	240	138.4	6.8	7.3	14.4
	280	106.0	42.2	6.8	14.7
	320	81.0	76.5	16.9	16.5
K	200	12.5	5.2	0.0	1.9
	240	97.5	5.0	2.4	11.1
	280	104.4	25.6	7.8	14.6
	320	73.5	72.0	16.6	17.1
Cs	200	0.4	0.0	0.0	0.0
	240	77.1	8.1	0.0	9.0
	280	90.5	18.3	4.2	11.9
	320	77.4	33.7	31.0	15.0

Table S25. Summary of skatole carboxylation yields for M_2CO_3/TiO_2 ($M = K^+$ or Cs^+) as a function of temperature by direct mixing of the heterocycle and dispersed carbonate. Conditions: 250 mg M_2CO_3/TiO_2, 1.5 mmol skatole, 4.0 bar CO_2 at 298.15 K, and 3 h reaction time.

M_2CO_3 /TiO_2	T (°C)	C2–CO_2^- (μmol/g TiO_2)	CH_2–CO_2^- (μmol/g TiO_2)	Other–CO_2^- (μmol/g TiO_2)	% CO_3^{2-} Conversion
K	200	9.9	0.0	0.0	1.0
	240	58.5	0.0	0.0	5.6
	280	83.6	19.9	9.9	10.9
	320	78.1	56.9	38.5	16.7
	360	45.3	23.6	36.3	10.1
Cs	200	1.6	0.0	0.0	0.2
	240	62.9	0.0	0.0	6.7
	280	85.8	39	22.7	15.7
	320	91.0	41.9	30.8	17.4
	360	24.1	6.1	10.4	4.3

Table S26. Summary of cesium benzothiophene-2-carboxylate/TiO_2 volatilization as a function of temperature. Reaction conditions: ~100 mg of supported carboxylate, 0.3 bar of dimethyl carbonate flowing at 40 mL/min for 120 min. The organics were isolated downstream using a cold trap while un-reacted supported carboxylates were isolated by aqueous extraction of the post-reaction TiO_2 support. Yields were determined by ^1H NMR using an internal standard.

T (°C)	Isolated Ester (μmol/g TiO_2)	Unreacted Supported Carboxylate (μmol/g TiO_2)	%Recovery (Ester + Carboxylate)
240	18.4	56.3	61
260	24.1	68.9	72
280	47.0	18.5	67
300	21.4	0.0	17
320	0.0	0.0	0

Table S27. Summary of temperature titrations for cesium benzothiophene-2-carboxylate/TiO_2 esterification reaction as a function of temperature for Cs. Reaction conditions: ~100 mg of dispersed carboxylate ($RCOOCs/TiO_2$) and 1 mL of dimethyl carbonate heated to specified temperature for 6 h under N_2 atmosphere. %Recovery is the sum of both unreacted, supported carboxylate and isolated ester. Ester was isolated by ethyl ether extraction while unreacted, supported carboxylate was isolated by aqueous extraction of the post-reaction TiO_2 support. Both carboxylate and ester were then quantified by ^1H NMR.

T (°C)	Ester Yield (μmol/g TiO_2)	Unreacted Supported Carboxylate (μmol/g TiO_2)	%Recovery (Ester + Carboxylate)
120	14.5	156.0	87
140	166.5	3.5	87
160	154.7	0.0	93
180	148.7	1.1	90
200	133.4	6.4	84
240	80.3	4.0	51
280	18.7	0.0	11

Table S28. Carboxylation/Esterification cycling results for both Cs and K_2CO_3/TiO_2 using benzothiophene as the substrate. Cycling conditions: carboxylation at 280 °C for 3 h, esterification at 160 °C for 1.5h (180 °C for K), regeneration at 200 °C for 3 h under reduced pressure (250 °C after cycle 3 and 250 °C for 6 h after cycle 4). Carboxylation yield was determined by aqueous extraction of a small amount (~50 mg) of post carboxylation $RCOOM/TiO_2$ material followed by ^1H NMR. Ester yield was determined by ethyl ether extraction of the post esterification reaction mixture followed by ^1H NMR. Any unreacted carboxylates were screened by aqueous extraction of a small amount (~50 mg) of post esterification $RCOOM/TiO_2$. In all cases no supported carboxylates were observed after the esterification.

Metal	Cycle #	Supported Carboxylate Pre-reaction (μmols/g TiO_2)	Isolated Ester (μmols/g TiO_2)	%Recovery
Cs	1	161.6	155.1	96.3
	2	153.5	146.9	95.7
	3	152.1	149.4	98.2
	4	148.6	143.2	96.4
	5	161.1	155.0	96.2
K	1	178.3	170.7	96.5
	2	61.6	58.0	94.2
	3	66.3	62.7	94.5
	4	95.8	90.8	94.7
	5	162.5	157.2	96.8

References

1. Frisch, M. J.; Trucks, G. W.; Schlegel, H. B.; Scuseria, G. E.; Robb, M. A.; Cheeseman, J. R.; Scalmani, G.; Barone, V.; Petersson, G. A.; Nakatsuji, H.; Li, X.; Caricato, M.; Marenich, A. V.; Bloino, J.; Janesko, B. G.; Gomperts, R.; Mennucci, B.; Hratchian, H. P.; Ortiz, J. V.; Izmaylov, A. F.; Sonnenberg, J. L.; Williams; Ding, F.; Lipparini, F.; Egidi, F.; Goings, J.; Peng, B.; Petrone, A.; Henderson, T.; Ranasinghe, D.; Zakrzewski, V. G.; Gao, J.; Rega, N.; Zheng, G.; Liang, W.; Hada, M.; Ehara, M.; Toyota, K.; Fukuda, R.; Hasegawa, J.; Ishida, M.; Nakajima, T.; Honda, Y.; Kitao, O.; Nakai, H.; Vreven, T.; Throssell, K.; Montgomery Jr., J. A.; Peralta, J. E.; Ogliaro, F.; Bearpark, M. J.; Heyd, J. J.; Brothers, E. N.; Kudin, K. N.; Staroverov, V. N.; Keith, T. A.; Kobayashi, R.; Normand, J.; Raghavachari, K.; Rendell, A. P.; Burant, J. C.; Iyengar, S. S.; Tomasi, J.; Cossi, M.; Millam, J. M.; Klene, M.; Adamo, C.; Cammi, R.; Ochterski, J. W.; Martin, R. L.; Morokuma, K.; Farkas, O.; Foresman, J. B.; Fox, D. J. *Gaussian 16 Rev. C.01*, Wallingford, CT, 2016.
2. Shen, K.; Fu, Y.; Li, J.-N.; Liu, L.; Guo, Q.-X., What are the pKa values of C–H bonds in aromatic heterocyclic compounds in DMSO? *Tetrahedron* **2007,** *63* (7), 1568-1576.
3. Chen, D.; Huang, F.; Cheng, Y.-B.; Caruso, R. A., Mesoporous Anatase TiO2 Beads with High Surface Areas and Controllable Pore Sizes: A Superior Candidate for High-Performance Dye-Sensitized Solar Cells. *Advanced Materials* **2009,** *21* (21), 2206-2210.
4. Xiao, D. J.; Chant, E. D.; Frankhouser, A. D.; Chen, Y.; Yau, A.; Washton, N. M.; Kanan, M. W., A closed cycle for esterifying aromatic hydrocarbons with CO2 and alcohol. *Nature Chemistry* **2019,** *11* (10), 940-947.
5. Yamamoto, M.; Osima, K.; Matsubara, S., Platinum Catalyzed H-D Exchange Reaction Of Various Aromatic Compounds Under Hydrothermal Condition. *Heterocycles* **2006,** *67* (1), 353-359.
6. Yang, L.; Liu, Z.; Li, Y.; Lei, N.; Shen, Y.; Zheng, K., Electrochemically Enabled C3-Formylation and -Acylation of Indoles with Aldehydes. *Organic Letters* **2019,** *21* (19), 7702-7707.
